# The evolutionary history and ancestral biogeographic range estimation of old-world Rhinolophidae and Hipposideridae (Chiroptera)

**DOI:** 10.1186/s12862-022-02066-x

**Published:** 2022-10-03

**Authors:** Ada Chornelia, Alice Catherine Hughes

**Affiliations:** 1 Landscape Ecology Group, Center for Integrative Conservation, Xishuangbanna Tropical Botanical Garden (XTBG), Chinese Academy of Sciences, Yunnan, People’s Republic of China; 2 International College, University of Chinese Academy of Sciences (UCAS), Huairou, Beijing, People’s Republic of China; 3 School of Biological Sciences, University of Hong Kong, Pokfulam, Hong Kong SAR People’s Republic of China

**Keywords:** Ancestral range, Dispersal, Horseshoe bats, Leafnosed bats, Oriental, Zoogeography

## Abstract

**Background:**

Family Rhinolophidae (horseshoe bats), Hipposideridae (leaf-nosed bats) and Rhinonycteridae (trident bats) are exclusively distributed in the Old-World, and their biogeography reflects the complex historic geological events throughout the Cenozoic. Here we investigated the origin of these families and unravel the conflicting family origin theories using a high resolution tree covering taxa from each zoogeographic realm from Africa to Australia. Ancestral range estimations were performed using a probabilistic approach implemented in BioGeoBEARS with subset analysis per biogeographic range [Old-World as whole, Australia–Oriental–Oceania (AOO) and Afrotropical–Madagascar–Palearctic (AMP)].

**Result:**

Our result supports an Oriental origin for Rhinolophidae, whereas Hipposideridae originated from the Oriental and African regions in concordance with fossil evidence of both families. The fossil evidence indicates that Hipposideridae has diversified across Eurasia and the Afro-Arabian region since the Middle Eocene. Meanwhile, Rhinonycteridae (the sister family of Hipposideridae) appears to have originated from the Africa region splitting from the common ancestor with Hipposideridae in Africa. Indomalaya is the center of origin of Rhinolophidae AOO lineages, and Indomalayan + Philippines appears to be center of origin of Hipposideridae AOO lineage indicating allopatric speciation and may have involved jump-dispersal (founder-event) speciation within AOO lineage. Wallacea and the Philippines may have been used as stepping stones for dispersal towards Oceania and Australia from the Oriental region. Multiple colonization events via different routes may have occurred in the Philippines (i.e., Palawan and Wallacea) since the Late Miocene. The colonization of Rhinolophidae towards Africa from Asia coincided with the estimated time of Tethys Ocean closure around the Oligocene to Miocene (around 27 Ma), allowing species to disperse via the Arabian Peninsula. Additionally, the number of potential cryptic species in Rhinolophidae in Southeast Asia may have increased since Plio-Pleistocene and late Miocene.

**Conclusion:**

Overall, we conclude an Oriental origin for Rhinolophidae, and Oriental + African for Hipposideridae. The result demonstrates that complex historical events, in addition to species specific ecomorphology and specialization of ecological niches may shape current distributions.

**Supplementary Information:**

The online version contains supplementary material available at 10.1186/s12862-022-02066-x.

## Background

Bats constitute the second most diverse group of mammals after rodents, with over 1400 species recognized to date [1]. The rapid diversification of this order may reflect their capacity for powered flight and echolocation, which has allowed them to colonize a wide range of ecological niches [2, 3], and is responsible for bats frequently represent the only native mammals on oceanic islands. In the Old World, insectivorous bats are split into those within the Yangochiroptera (of which most families are not limited to the Old World), and the Old-World endemic Yinpterochiroptera, which includes Rhinolophoidea as well as the frugivorous Pteropodidae, (with Old-World insectivorous bat communities frequently dominated by Rhinolophoidea). Here, we focus exclusively on Old-World insectivorous bats, the superfamily Rhinolophoidea and to date, it encompasses six families of insectivorous bats (Crasseonycteridae, Megadermatidae, Rhinopomatidae, Rhinolophidae, Hipposideridae and Rhinonycteridae [2, 4], but in this study we only focus on the three latter families due to their high diversity (Crasseonycteridae only has one described species, and Megadermatidae only six species). Rhinolophidae, Hipposideridae and Rhinonycteridae are monophyletic and are closely related, with Hipposideridae as sister family of Rhinolophidae and Rhinonycteridae recently resolved as separate family from Hipposideridae.

Rhinolophidae (horseshoe bats) consist of a single genus *Rhinolophus* Lacépède, 1799. They are insectivorous bats distributed throughout the Old World, primarily in tropical regions, from Africa through Eurasia, Oceania and Australia [5]. Around 106 species have been described to date [1], although this number is likely to be an underestimate given that many species are cryptic [6]. The common name of Rhinolophidae is derived from specialized horseshoe-shaped noseleaf which is used to emit acoustic calls emission [7–13]. The distinctive noseleaf morphological components, include the sella, lancet, furrows of lancet, internarial cup and ears shape of each rhinolophids species plays important role in determining call structure, and may provide useful cues in the identification of cryptic species [6].

Hipposideridae is the sister family of Rhinolophidae. Commonly known as leaf-nosed bats, they are distributed in the same range as Rhinolophidae across the Old World [14]. They consist of seven genera: *Hipposideros*, *Anthops*, *Asellia*, *Aselliscus*, *Coelops*, *Doryrhina*, *Macronycteris*, including 90 described species [1]*.* Recently *Cloeotis*, *Paratriaenops*, *Rhinonycteris*, and *Triaenops* were elevated to a separate family, Rhinonycteridae (the Trident bats) [15], and is sister family to Hipposideridae [14]. *Hipposideros* is the most diverse genus within Hipposideridae, which includes almost 80% of the total species in the family, and many species are cryptic [13, 16, 17], and thus true diversity may be considerably higher [1].

The systematics of these groups have not been well resolved. Although recent years have seen significant progress towards resolving the systematics of bats using an abundance of phylogenetic datasets [4], these analyses generally had low systematic coverage and have only included a limited number of species within each family, and with limited spatial coverage [14, 18]. As a result, the understanding of systematics and evolution in these predominantly tropical groups are limited by the number of genes in the study, and systematic biases from limited taxon sampling and low geographical coverage [14, 18–25]. As the consequence of contradictory dating estimations, the interfamilial time divergence estimation within Rhinolophidae and Hipposideridae still has not been reliably estimated, thus assessing the evolutionary history and the biogeographic origin is still challenging (i.e., [14, 18, 22–25].

Accurate inferrence of the biogeographic history of a taxon is heavily dependant on estimated ages and relationship between taxa, but often lacks rigour when it is presented as a narrative in addition to phylogenetic studies without explicit analysis [26–28]. The geographic origin of ancestral Rhinolophidae remains unresolved due to the limited fossil record, lack of representative taxonomic sampling across their distribution [14], and conflicting phylogenies [20].

The biogeographic history of bats is particularly interest due to their capability of true flight and ability to disperse over a wide geographic area. Several alternative hypotheses of the Rhinolophidae family origins have been proposed based on different data types and methods, for instance European origin [29], Asian origin [18, 23, 30], African origin [14, 22], and Middle-Eastern origin [20]. Flight ability relates to wing shape for each species and thus has direct implications for the probability of these routes (as reviewed in Norberg and Rayner 1987) [2, 31–33]. Bats with pointed wings and a high aspect ratio are expected to have energetically inexpensive flight and be able to travel long distances but have low manouverability. Wings with high wing loading enables fast flight to reduce the time invested to achieve long distance flight, thus suitable for migration and dispersal over long-distances [34]. Conversely, broad-short wings with rounded tips (low wing loading, low aspect ratio) which are present through most rhinolophids species may limit dispersal of species across large water bodies or other open areas [35] and current distribution of taxa may have follow vicariance events in the past. Furthermore, different methods have different historical biogeographic event assumptions, which has a significant influence on the inferences. Thus, to distinguish between these competing hypothesis, testing and comparison between statistical approaches is needed to optimize model selection and conduct meaningful and well-supported biogeographic analyses [36].

**Fig. 1:**
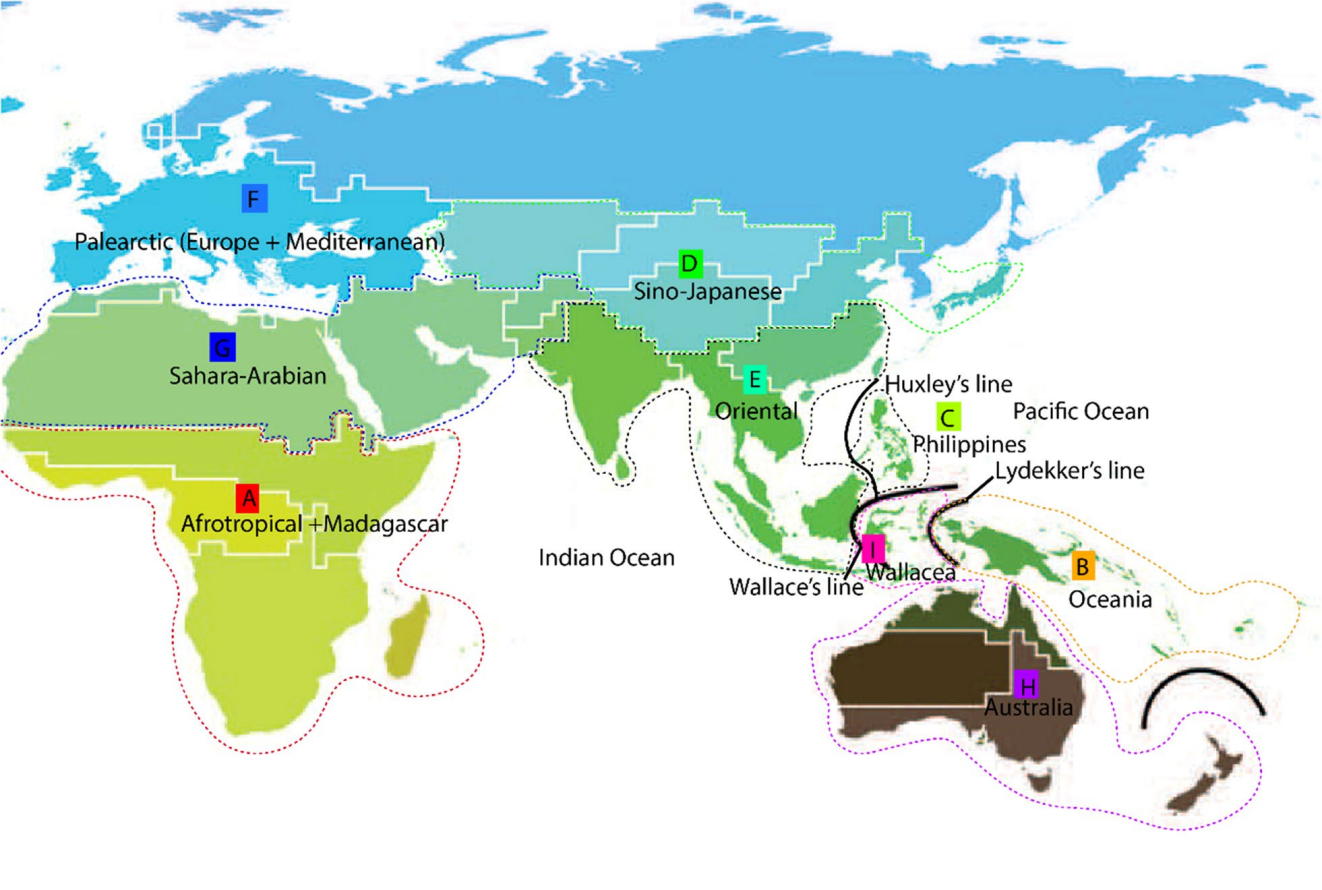
The map of terrestrial zoogeographic and regions of the Old-World tropic, modified from Holt et al. (2013). Dashed lines indicate figurative borders between zoogeographic regions which we used for Rhinolophoidea superfamily analysis (Rhinolophidae, Hipposideridae and Rhinonycteridae). Bold lines indicate Wallaces Line, Huxleys Line and Lydekkers Line

In this study, we use probabilistic modeling of geographic range evolution, which allows users to statistically choose the number of biogeographic models based on Maximum Likelihood and Bayesian methods implemented in the R package “BioGeoBEARS” [36–39]. However, to date, no study has performed this analysis in Rhinolophidae and Hipposideridae. This approach unifies multiple models and provides a flexible framework for comparing alternative models in a statistical context. Here we use representative species distributed in each biogeographic realm (Fig. 1), ranging from the Afrotropical, Palearctic (Europe and Mediterranean), Oriental, to Oceanic and Australian realms. To the best of our knowledge, this study is the first to infer the ancestral ranges of Rhinolophidae and Hipposideridae using explicit biogeographic analysis. In addition, here we attempt to infer the evolutionary history and biogeographic ranges of potential cryptic species of Asian Rhinolophidae (revised in [6]), in particular Southeast Asian region. We aim to understand historical biogeography of a subset of the superfamily Rhinolophoidea (Rhinolophidae, Hipposideridae and Rhinonycteridae) of Old World and the evolutionary history of potential cryptic species of Rhinolophidae.

## Results

### Historical biogeography of Rhinolophoidea superfamily

**Fig. 2:**
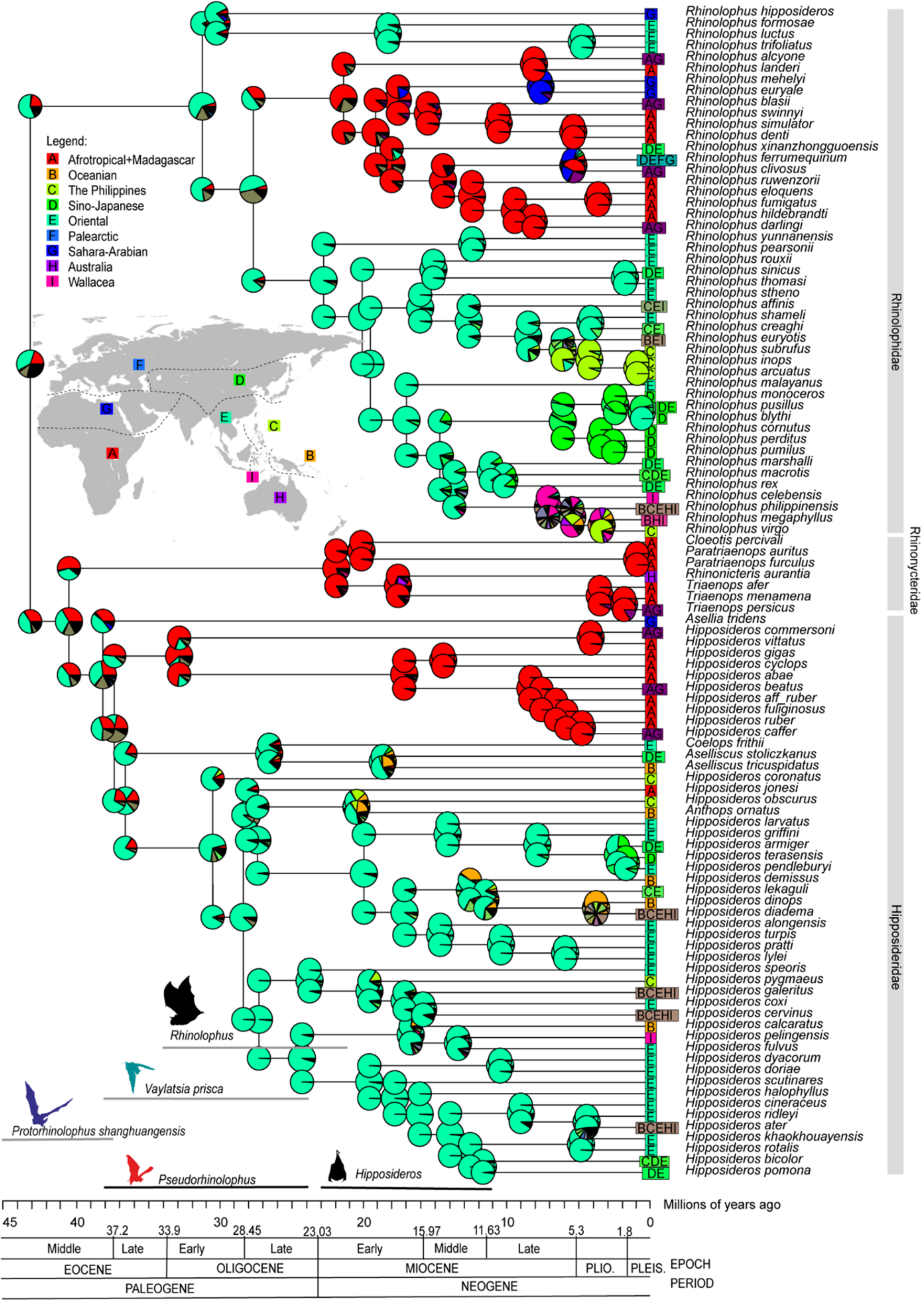
Ancestral range estimation for superfamily Rhinonophoidea (Rhinolophidae, Hipposideridae and Rhinonyteridae (n = 104 extant species)). Tree topology were pruned from Álvarez-Carretero et al. (2021). Pie-charts indicate the relative probability of each state in the nodes and corners. The corners represent the states of the descendant lineage instantaneously after speciation and each geographic range represent in encoded colors (See inserted maps and legends). The inserted figure in the lower part represents the fossils of ancestoral *Rhinolophus* and *Hipposideros*, the barline below the species name indicates the timescale scored after Branch and Bound analysis, (adapted from Ravel et al. 2016). Bat silhoutte images of extinct species were downloaded from PhyloPic (http://phylopic.org/) CC-SA

The time-tree pruned from Álvarez-Carretero et al. (2021) is provided in Additional file 1: Fig. S1, further, the time divergence estimations mentioned in this result were cited based on Álvarez-Carretero et al. 2021 study. In general, our result showed Likelihood Ratio Test (LRT) using chi-squared one-tailed test conferred higher log likelihood (natural log) for + *j* models (Table 1). However, the likelihood ratio test (LRT) shows there was no significant difference between DEC* + J and its nested models, DEC* (Lnl = − 302.6891; Lnl = − 302.6879, respectively, *p* = 1), shown the two models has the same likelihood on the data. While the DEC* + J model for Rhinolophoidea has some AIC support, its estimate of *j* is almost 0, therefore there is no support of founder events being important in this clade. To ensure the same likelihood in DEC* and DEC* + J was not due to the maximum likelihood optimization problems and computational issues, we ran the analysis three times and similar results were acquired. Here we focus on reporting the result under the best-fit model given the data, less-fitted models are provided in Additional file 1: Figs. S2–S9. The best fit model given the data is DEC* and followed with DEC* + J (Table 1). In DEC* model, extinction *e* value is higher than dispersal *d* (*d* < *e*; *d* = 0.0078; *e* = 0.0685) indicating the range contraction rate is higher than range expansion event in this superfamily (Fig. 2).

**Table 1:** Best-fit models based on BioGeoBEARS analysis (all models statistical results are provided for Superfamily Rhinolophoidea but only best-fit models provided for the rest of analyses)

	LnL	np	*d*	*e*	*j*	AICc	AICc_wt
Superfamily Rhinolophoidea							
DEC	− 320.09	2	0.00457	0.00024	NA	644.346	2.07E−08
DEC + J	− 315.26	3	0.00408	0	0.01096	636.871	8.71E−07
DIVALIKE	− 339.08	2	0.00576	0.00335	NA	682.321	1.18E−16
DIVALIKE + J	− 331.65	3	0.00465	0	0.01275	669.634	6.70E−14
BAYAREALIKE	− 390.63	2	0.01	0.01	NA	785.426	4.81E−39
BAYAREALIKE + J	− 331.12	3	0.00328	0	0.02453	668.577	1.14E−13
DEC*	− 302.69	2	0.00782	0.06852	NA	609.545	0.74802
DEC* + J	− 302.69	3	0.00782	0.06846	NA	611.721	0.25198
Rhinolophidae: Australia–Oriental–Oceanian (AOO) lineage							
DEC*	− 90.569	2	0.0156	0.04445	NA	185.308	0.71898
DEC* + J	− 90.56	3	0.015	0.038	0.0028	187.1	0.26
Rhinolophidae: Afrotropical–Palearctic lineages							
BAYAREALIKE	− 56.893	2	0.01371	0.04275	NA	117.955	0.71729
BAYAREALIKE + J	− 56.89	3	0.014	0.043	0	120.1	0.24
Hipposideridae: Oriental–Australia lineages							
DEC + J	− 150.61	3	0.00611	0	0.02877	307.558	0.47514
DEC*	− 151.9	2	0.01	0.01	NA	307.965	0.38752
DEC* + J	− 151.88	3	0.01	0.01	NA	310.111	0.13252
Hipposideridae and Rhinonycteridae: Afrotropical–Palearctic lineages							
DEC*	− 58.64	2	0.54138	2.43769	NA	121.448	0.78792
DEC* + J	− 58.91	3	0.21	0.84	0.44	124.22	0.20
Cryptic Rhinolophidae							
DEC*	− 197.2	2	0.041	0.67	NA	398.6	0.19
DEC* + J	− 194.7	3	0.021	0.298	0.023	395.708	0.81136

*LnL* Log-likelihood, *np* number of parameters, *d* dispersal, *e* extinction, *j* jump dispersal, *AICc* Akaike Information Criterion (corrected), *AICc_wt* AICc weighted

Our result suggests that the common ancestor of Rhinolophoidea superfamily as a whole [Rhinolophidae, (Hipposideridae, Rhinonycteridae)] were from the Oriental region (Fig. 2), diverged around middle Eocene at 43.25 Ma (95% highest posterior density/HPD = 39.74–46.76 Ma) with the percentage of relative probability was slightly higher from Oriental region than the model which showed an origin in the Afrotropical region (Fig. 2). Similarly, more than 50% relative probability showed that the ancestor of Rhinolophidae family also from Oriental region, and then later expanded to colonized Afrotropical region. The common ancestor of Hipposideridae and Rhinonycteridae were suggested to be from Oriental and Afrotropical regions (with almost the same relative probability for each region but slightly higher from Oriental), where Rhinonycteridae split earlier around middle Eocene at 40.55 Ma (95% HPD = 36.03–44.14 Ma) from the rest of Hipposideridae of Afrotropical lineages (Fig. 2).

### Historical biogeography of family Rhinolophidae (Horseshoe bats).

The Rhinolophidae family split into two major lineages in the early Oligocene at 27.68 Ma (95% HPD = 23.96–31.54 Ma): *Rhinolophus* Afrotropical–Palearctic lineages and Oriental lineages (with Australian and Oceanic species). *R. trifoliatus*, *R. luctus*, *R. formosae* and *R. hipposideros* are sisters with respect to the above two lineages, and split earlier in the early Oligocene at 31.24 Ma (95% HPD = 27.36–35.16 Ma). The best fit model given the data was DEC* model followed with DEC* + J (Table 1). Similarly with Rhinolophoidea superfamily, range expansion is lower than extinction (*d* < *e*; *d* = 0.0156, *e* = 0.0444) suggesting that range constriction is higher compared to dispersal rate in historical colonization Rhinolophidae.

**Fig. 3:**
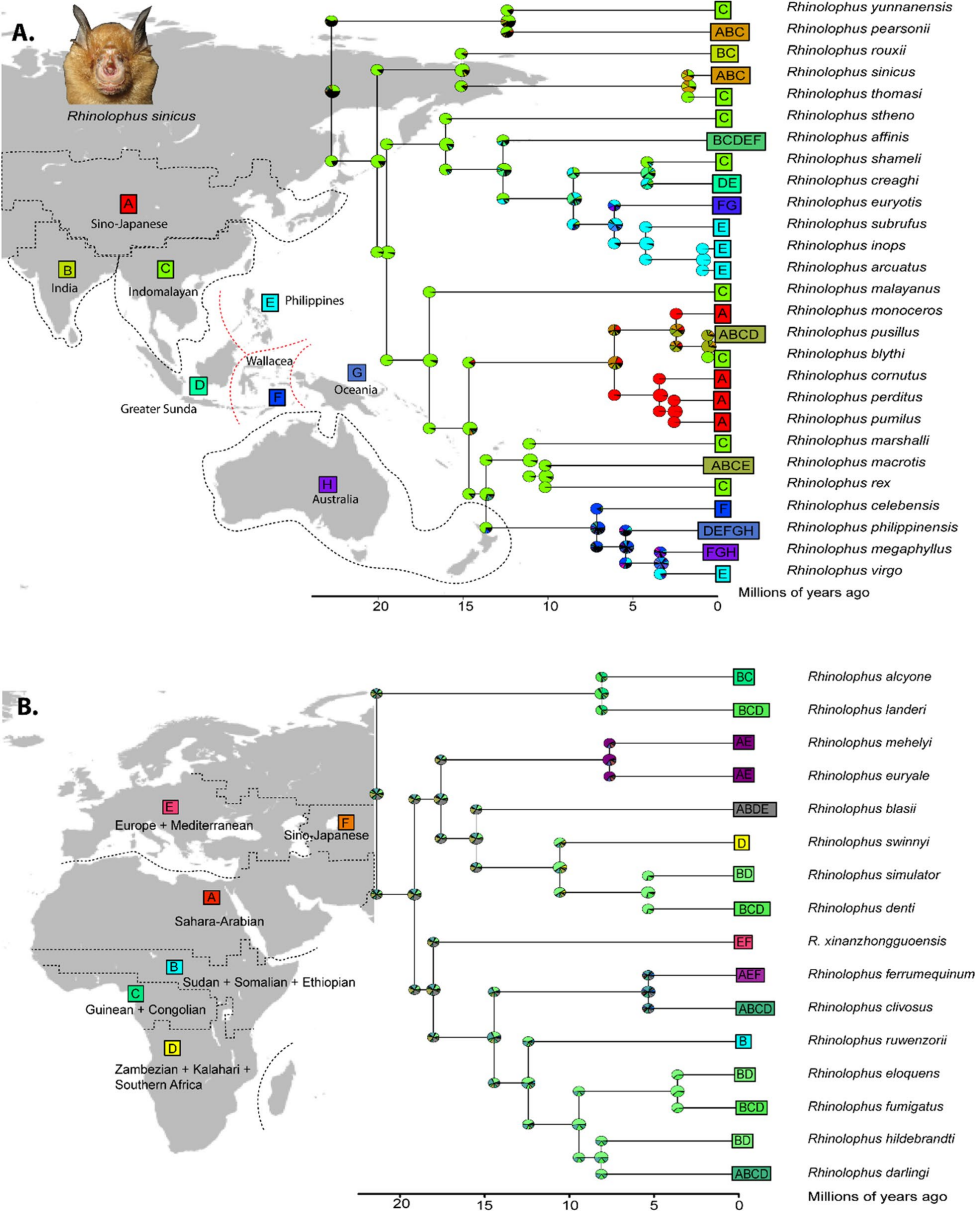
Ancestral range estimation for Rhinolophidae. **a** Oriental–Oceanian–Australia region; **b** Afrotropical-Palearctic (Europe + Mediterranean) region. Inserted maps are adapted from Holt et al. 2013. [Insert photo, not to scale: *R. sinicus* (photo by Ada Chornelia)]

The result from DEC* suggests that ancestor of Rhinolophidae species the Oriental–Oceania–Australia lineage originated from Indomalayan region and species diversification began around Early Miocene around 22.77 Ma (95% HPD = 19.25–26.51 Ma) (Fig. 3). The ancestral range expanded from the Indomalayan region to the north to Sino-Japanese and west to India (within Asia continent) then later colonized towards the Philippine archipelago around end of Middle Miocene at 12.64 Ma (95% HPD = 9.37–16.19 Ma) (Fig. 3). The result shows ancestral Japanese rhinolophids originated from the Indomalayan region, the species thus diversified around the Middle Pliocene at 3.43 Ma (95% = 1.75–5.67 Ma) with high endemicity of extant species such as *R. cornutus*, *R. pumilus*, and *R. perditus* (Fig. 3a). The rhinolophid diversification in the Philippine archipelagoes is estimated to have occurred around the Late Miocene–Early Pleistocene at 6.09 Ma (95% HPD = 3.31–4.53 Ma). The Philippine rhinolophids appears to be the result from multiple colonization events originating from the Indomalayan and Wallacean regions. Rhinolophids colonization in Wallacea region, also occurred in multiple colonization event from the Indomalayan region through greater Sunda islands and Philippine archipelago, around Late Miocene at 7.12 Ma (95% HPD = 4.46–10.2 Ma). Australian and Oceanian rhinolophids appears to colonized from the Indomalayan region through two pathways, from the Indomalayan region–Sundaland–Wallacea and Indomalayan–Philippine–Wallacea which occurred in Late Miocene to Early Pliocene (Fig. 3a).

Moreover, the Afrotropical–Palearctic Rhinolophidae diversified around Early Miocene at 21.38 Ma (95% HPD = 17.74–25.36 Ma), the result exhibit BAYAREALIKE has 72% being the best fit model given the data, and ancestral range contraction rate is higher than dispersal or range expansion (*d* < *e*; *d* = 0.0137; *e* = 0.042755) (Table 1). BAYAREALIKE assumes the ancestral range process is predominantly explained by sympatry (subset and widespread) and no vicariance events occured. It is important to note that the relative probabilities in the nodes and the corner showed less than 50% probabilities for each states. Therefore, the ancestral species may be widely distributed in most of Afro-Palearctic region including Europe-Mediterranean, Sahara-Arabian, Sudanian-Somalia-Ethiopia, Zambezian-Kalahari-Southern Africa region (ABDE, Fig. 3b), or the combination of these regions. The ancestral ranges of Rhinolophidae predominantly occupied Sudanian + Somalia + Ethiopia region and Zambezian + Kalahari + Southern Africa around Middle Miocene to Pliocene (15–3.62 Ma). Re-colonization to Sahara-Arabian and Europe-Mediterranean might have occurred around Late Miocene at 7.63 Ma (95% HPD = 3.9–12. 14 Ma) (Fig. 3b).

### Historical biogeography of family Hipposideridae (leaf-nosed bats) and Rhinonycteridae (the Trident bats)

**Fig. 4:**
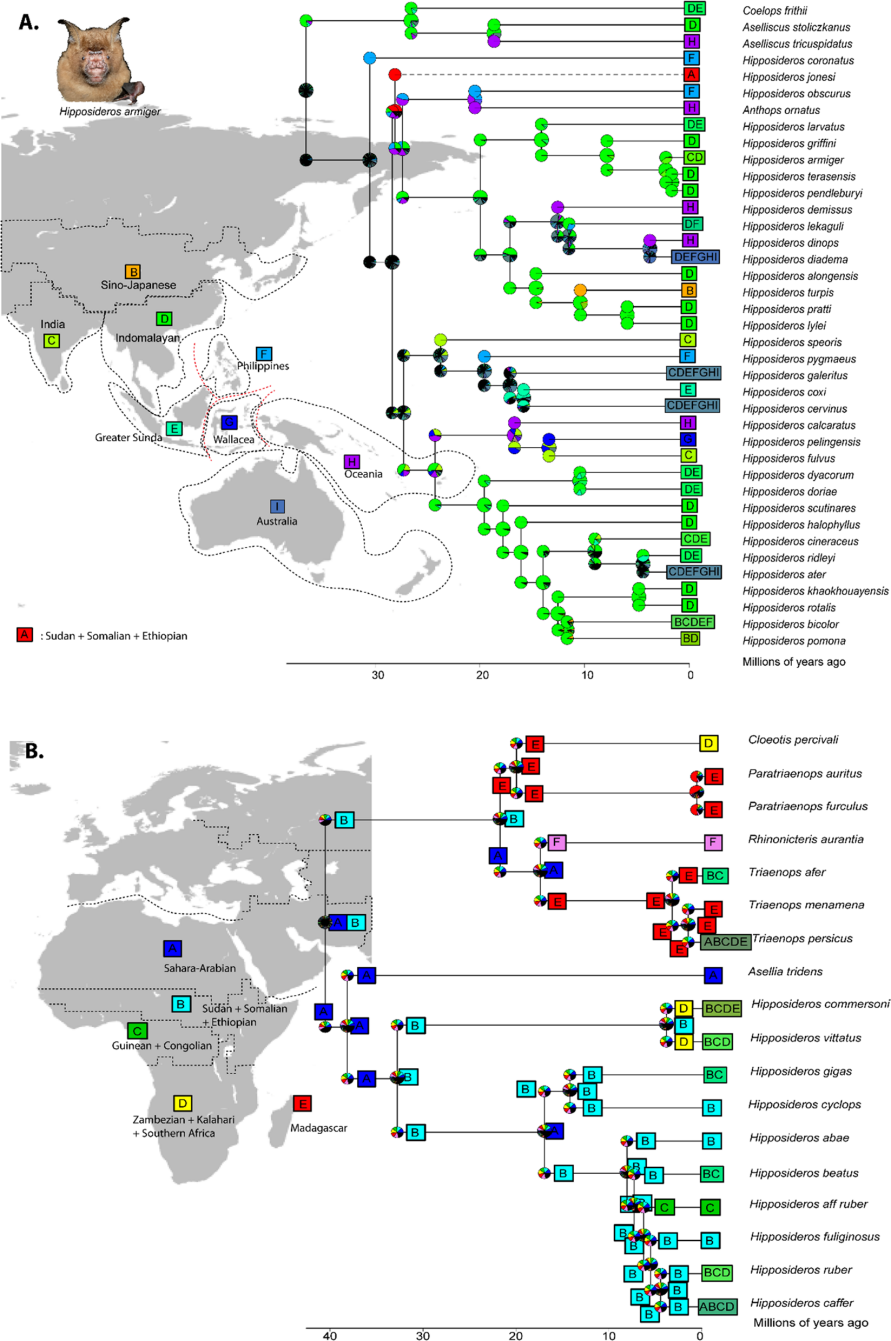
Ancestral range estimation for **a** Hipposideridae of Oriental–Oceanian–Australia region; **b** Hipposideridae and Rhinonycteridae of Afrotropical–Palearctic (Europe + Mediterranean) and Madagascar region. Inserted maps adapted from Holt et al. 2013. [Insert photo, not to scale: *H. armiger* (photo by Ada Chornelia)]

The ancestors of the Hipposideridae family appears to have a wide distribution from the Oriental to the African region (see above results). The subset analysis results showed that DEC + J has a 47% probability of being the best fit model in the hipposiderids Oriental–Oceania–Australian lineages, alternatively, the other two models had strong support: DEC* (39% probability) and DEC* + J (13% probability). All trees generated in these models generally recovered the same ancestral range for each node, therefore indicated the similar scenarios. The single-most probable states in the output of the three models indicated Indomalayan + Philippines origins, however the relative probabilities given in early ancestral ranges were poorly supported (Fig. 4a). The result showed the dispersal rate is higher than range contraction (*d* > *e*; *d* = 0.00611,* e* = 0, *j* = 0.0288), which is in contrast with Rhinolophidae family, where dispersal rate is smaller than extinction rate. The hipposiderids ancestral range then expanded to India, Greater Sunda Islands, Wallacea and Oceania in the early Oligocene (30.52 Ma (95% HPD = 25.26–34.92 Ma). Colonization towards the Greater Sunda islands was directly from the Indomalayan region which occurred since the early Miocene. Oceanian hipposiderids appears to have colonized the region since early Oligocene and may use the Philippines as a stepping stone. The hipposiderids in Wallacea and Australia regions may have been colonized through greater Sunda Islands, as this coincided with historical presence of land bridges since Oligocene to Pliocene (Fig. 4a). Paraphyly within Hipposideridae is shown in African species *H. jonesi* (A = Sudanian + Somalia + Ethiopia) which was recovered within the rest of Oriental–Oceania–Australian taxa, diverged around early Oligocene at 28.12 Ma (95% HPD = 24.45–31.82 Ma), which may indicated earlier Asian–African colonization.

Furthermore, we found that DEC* has 79% probability of being the best fit model for Hipposideridae and Rhinonycteridae Afrotropical lineages, followed by DEC* + J (20% probability) with range contraction is higher than range expansion (*d* < *e*; *d* = 0.54,* e* = 2.43) (Table 1). The result suggested that the ancestral of Hipposideridae Afrotropical lineage were from the Sahara-Arabian (A) and Sudan-Somalian-Ethiopian (B) regions (Fig. 4b). The diversification of Hipposideridae Afrotropical lineages began around middle Eocene with splitting of *Asellia tridens* with the rest of *Hipposideros* genera (38.18 Ma (95% HPD = 36.93–41.79 Ma), originated from Sahara-Arabian region (however with little percentage of relative probability, Fig. 4b). Multiple colonization occurred to Guinean-Congolian region via range expansion from the Sahara-Arabian region through Sudanian + Somalia + Ethiopia and from Southern part (Zambezian + Kalahari + Southern Africa). Madagascar Hipposideridae ancestral originated from African continent (Fig. 4b). Similarly, the ancestral of Rhinonycteridae may have originated from Africa continent then colonized Madagascar which began at early Miocene at 21.88 Ma (95% HPD = 16.54–28.48 Ma). Our result suggests over-land dispersal event might occurred to expand Rhinonycteridae ancestral range from the Africa continents to Madagascar, however, it is necessary to consider that each of ancestral state in the result of this lineage were having almost similar relative probability percentage for each state and we discussed below.

### The evolutionary history and historical biogeography range of potential cryptic species of Rhinolophidae Asia lineages

**Fig. 5:**
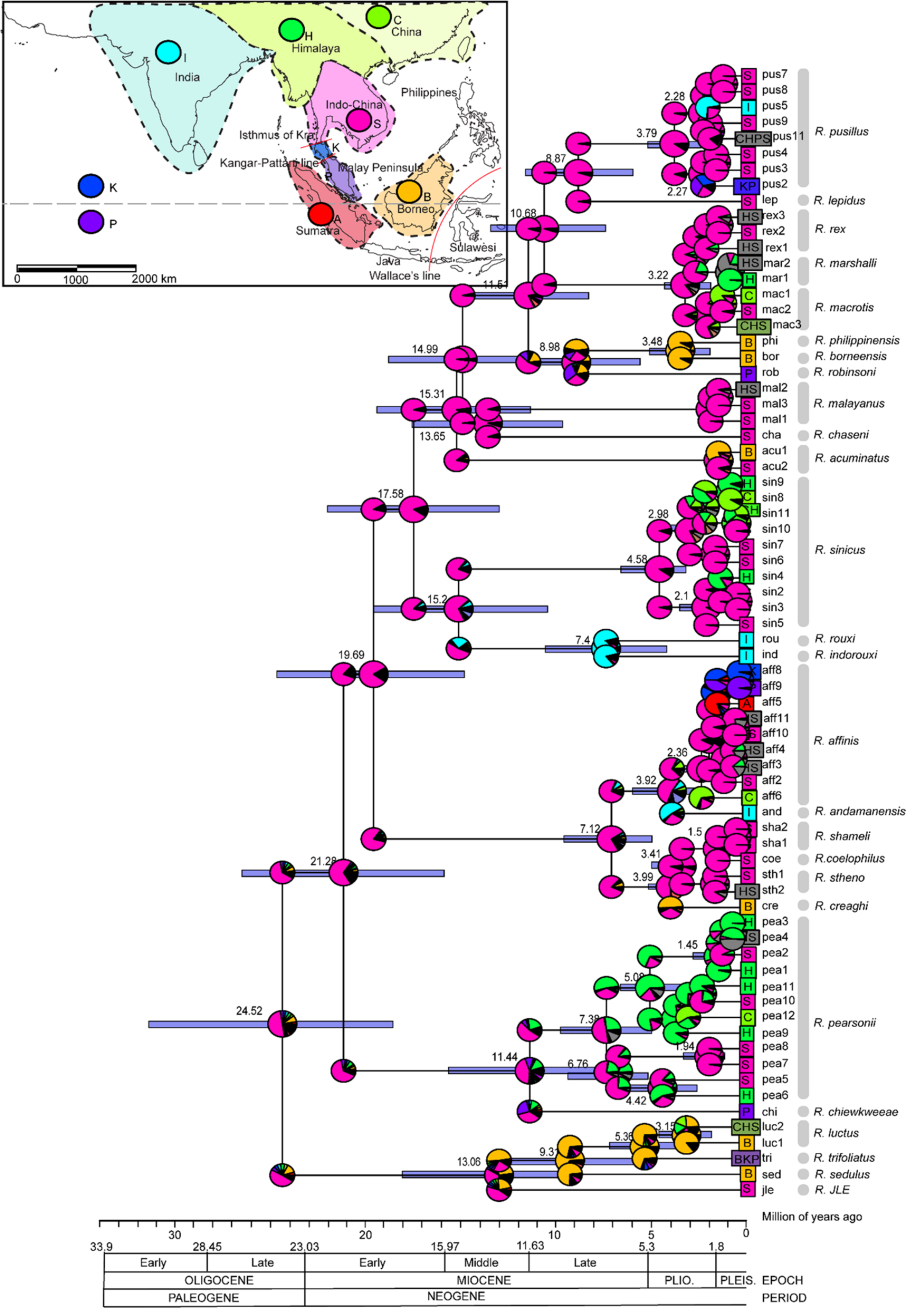
Historical biogeography of Rhinolophidae in Southeast Asia with representation of potential cryptic species. Date estimation with 95% Highest Posterior Density (HPD) are provided in the nodes generated from BEAST. Current distribution of extant taxa based on the distribution of potential cryptic species and sampling included in the study. Each potential cryptic species is given unique identifiers (based on Chornelia et al. 2022)

The result showed the estimation of time divergences between Rhinolophidae and Hipposideridae began in late Eocene, with posterior age 40.26 Ma (95% HPD = 37–43.5 Ma), slightly younger but in general fall within the dating range estimation in previous studies as 42 Ma in [14], 45.47 Ma in [4] and 43 Ma (95% HPD = 39–46 Ma) in [43]. Our analysis showed the diversification of Rhinolophidae in the region began around late Oligocene till early Miocene. *R. JLEsp* (undescribed species [69]; the acronym from [70] denoting Judith L. Eger), *R. sedulus*, *R. trifoliatus* and *R. luctus* represent the oldest lineages, separated from other *Rhinolophus* species around 24.52 Ma (95% HPD = 16.5–27 Ma), in concordance with Álvarez-Carretero et al. (2021) positioned *R. luctus*, *R. trifoliatus* and *R. hipposideros* (not included in this analysis) as the sister of Rhinolophidae species but their estimation is higher when Rhinolophidae Afrotropical lineages is included as of 31.24 Ma (95% HPD = 27.36–35.16 Ma)). Rapid speciation and species diversification in Indochina region happened approximately since late Miocene (23 Ma) (Fig. 5), coincided with date estimated from with Álvarez-Carretero et al. (2021) [22.77 Ma (95% HPD = 19.25–26.51 Ma)]. The majority of potential cryptic species or incipient species within rhinolophids species complexes diverged in mid-Pliocene within the last 2 Ma indicates rapid radiation in Plio-Pleistocene, with the exception of the *R. pearsonii* complex that diverged during mid-Miocene 7.38 Ma (HPD = 5.2–9 Ma) indicated a support to split *R. pearsonii* into multiple different species. The time divergence construction using secondary calibration at multiple nodes in BEAST supported the evolutionary distinction between the clades within species complexes and the incipient species might further be considered as distinct species.

The result of historical geographic ranges estimation for cryptic Rhinolophidae suggests DEC* + J has 82% probability of being the best fit model given the data and DEC* (Dispersal–Extinction–Cladogenetic) has 18% probability of best fit model, and other models shown < 1% of probabilities as fit model given the dataset. DEC* + J showed the estimate extinction rate (range contraction) events was higher compared to dispersal (range expansion) and jump-dispersal events (*d* < *e*; *d* = 0.0205, *e* = 0.298, *j* = 0.0228).

Cryptic Asian Rhinolophidae likely originated from the Indomalayan region, here in particular Indochina (Fig. 5), in concordance with our result in ancestral biogeographic of Rhinolophidae Oriental–Oceanian–Australian lineages, then expanded to current geographic ranges subsequently. The differences in the results we present here with other section (Rhinolophidae Oriental–Oceanian–Australian lineage) was the biogeographic areas and systematic coverages we used in the analysis (see “Materials and methods”).

The origin of *R. luctus*, *R. sedulus* and *R. trifoliatus* ancestors were in the Indomalayan region then occupied Borneo island via vicariance events since middle Miocene (13 Ma). Similarly, colonization to Borneo Islands from Indochina also present in *R. philippinensis* and *R. borneensis* around Pliocene (2–5 Ma). Our result suggests a rapid diversification began in the late Miocene around 7 Ma within *R. pearsonii* as the ancestral group where the ancestral ranges subset-sympatry into two largely ranges; some remained in Indochina and then subset-sympatry in northern areas (Himalaya region and China).

Our analysis indicates a high probability of recent diversification through subset-sympatry in *R. affinis* and *R. pusillus* group around Isthmus of Kra, Kangar-Pattani line to the tip of Malay Peninsula from the rest of northern population. Genetic drift followed by isolation and divergence may explain the emergence of separate geographical ranges of *R. affinis* in Sumatra Island after separation from mainland which shows a closer relationship to Isthmus of Kra, Kangar Pattani and Malay Peninsula lineages dated around the Pleistocene (2 Ma) (Fig. 5).

## Discussion

The ancestral range estimation for superfamily Rhinolophoidaea [Rhinolophidae, (Hipposideridae, Rhinonycteridae)] suggests that the ancestors of family were had an Oriental origin, then diversified to other biogeographic regions. African region were colonized through Sahara-Arabian regions, ancestral range expanded to Madagascar. The Oriental region experienced complex colonizations related to geological events in the region, including colonization toward Philippines, Indonesia (Greater Sunda Islands, Wallacea) and Oceania. Australian rhinolophoids may have colonized through the Philippine, Greater Sunda Islands, Wallacea and Oceania. Our results are congruent with the previous hypothesis that Rhinolophidae bats may have originated from the old world tropics in Asia [18, 23, 30, 73] then the range expanded to East Asia region including India, Africa and islands in the vicinity, but, in contrast with [14, 22] which hypothesized that the ancestral of *Rhinolophus* species were of African origin based on LAGRANGE biogeographic analysis. Rhinolophidae of Oriental–Oceanian–Australian lineages originated from the Indomalayan region, and the ancestral range of Afrotropical lineages were widespread in the Afro-Palearctic. Allozyme variability also suggests colonization from Eurasia toward North Africa and that subsequent diversification took place in Africa [74], and morphological studies suggest that plesiomorph Oriental rhinolophids were basal and Afro-Palearctic species were more derived [30]. The widespread ancestral and current distribution of taxa in best-fit model BAYAREALIKE indicated the ancestral ranges were similar to those occupied by their descendants, and suggests over-land range expansion in the past around Late Oligocene coincided with diversification of Afrotropical lineages at 27.68–24.38 Ma. The result showed that dispersal rate was constantly lower than extinction rate in Rhinolophidae (*d* < *e*). Similarly with Hipposideridae–Rhinonycteridae Afro-Palearctic lineages (*d* < *e*), but in contrast with Hipposideridae Oriental lineages where dispersal or range expansion is higher (*d* > *e*). The estimated rate of dispersal is low suggesting that species have mostly retained the same geographic ranges as their ancestors [36, 38].

The common ancestor of Hipposideridae originated from Oriental or Afrotropical regions (similar posterior probability, Fig. 2), suggesting possible widespread ancestral ranges of this family. The ancestor of Hipposideridae Oriental–Oceania–Australian lineages originated from the Indomalayan region and the Philippines, which suggests multiple colonizations may occured during range expansion. The common ancestors of Hipposideridae Afro-Palearctic lineages originated from Sahara-Arabian and Sudanian–Somalia–Ethiopia suggested the early colonization from northern part of Africa and Arabian Peninsula (Fig. 4b). Even though the inferences of ancestral ranges of Hipposideridae lineages are not strongly supported, but the results are in agreement with previous studies based on fossils which suggest that major the dispersal axis of Hipposideridae was from North Africa toward South Europe during the Middle Eocene [75]. The result also suggests that Rhinonycteridae in Madagascar split from their common ancestor (Hipposideridae Afrotropical lineages) around Middle Eocene (~ 40.55 Ma) and ancestral range originated from the Sudanian–Somalia–Ethiopia regions.

Our result supports the importance of statistical model testing as each model has strong assumptions which impact on inferences, as highlighted in [36]. Although, recent results by [71] revealed potential statistical problem for the comparison between DEC and DEC + J models and to alleviate this issue they proposed the use of geographic state speciation and extinction models (ClaSSE) to study range evolution. However, [72] argues that DEC and DEC + J can be compared using likelihood and AIC, and that the comparison is equivalent to ClaSSE comparisons. Despite these controversies, our results showed that model with + J were not always selected as the best fit models and the simulation results in [72] indicated better fit models will result in more accurate inference of parameters and ancestral ranges. However, it also important to consider that biogeographic range estimation can be biased by model choice.

Even thought our result indicates an Oriental (Indomalayan) origin for the Rhinolophidae, there is lack of Paleogene fossil found in the Indomalayan region, though variable taphonomy means that the majority of the fossil record is missing across taxa. The only known possible record of Eocene bats in this region is Megachiroptera from Krabi Mine in Thailand. However, fossil evidence of bats is relatively rare due to delicate skeletons and are therefore rarely preserved, thus, leaving only teeth and postcranial fragments for identifcation [76]. Fossils of ancestral Rhinolophidae include *Protorhinolophus shanghuangensis* from Shanghuang fissure, Jiangsu (northern part of Asia) [73], highlighting ancient distrobution in the Oriental region. The oldest bat fossils are from the early Eocene, and are known from North America, Europe, Africa and Australia [76, 77]. However, geographic origin of bats ancestor still being debated with two hypothesis of Laurasia or Gondwana origin [78]. The initial explosive radiation of bats occurred in Eocene, the extinct families such as “Eochiroptera” sensu Van Valen (1977) found in most of continents except Antartica [25, 77]. Modern radiation of extant taxa appears to have begun at least by Middle Eocene or earlier, a period characterized by a significant global rise temperature after K-Pg (Cretaceous–Paleogene) mass extinction event [19, 76, 79], which coincided with 43.25 Ma divergence between Hipposideridae and Rhinolophidae.

**Fig. 6:**
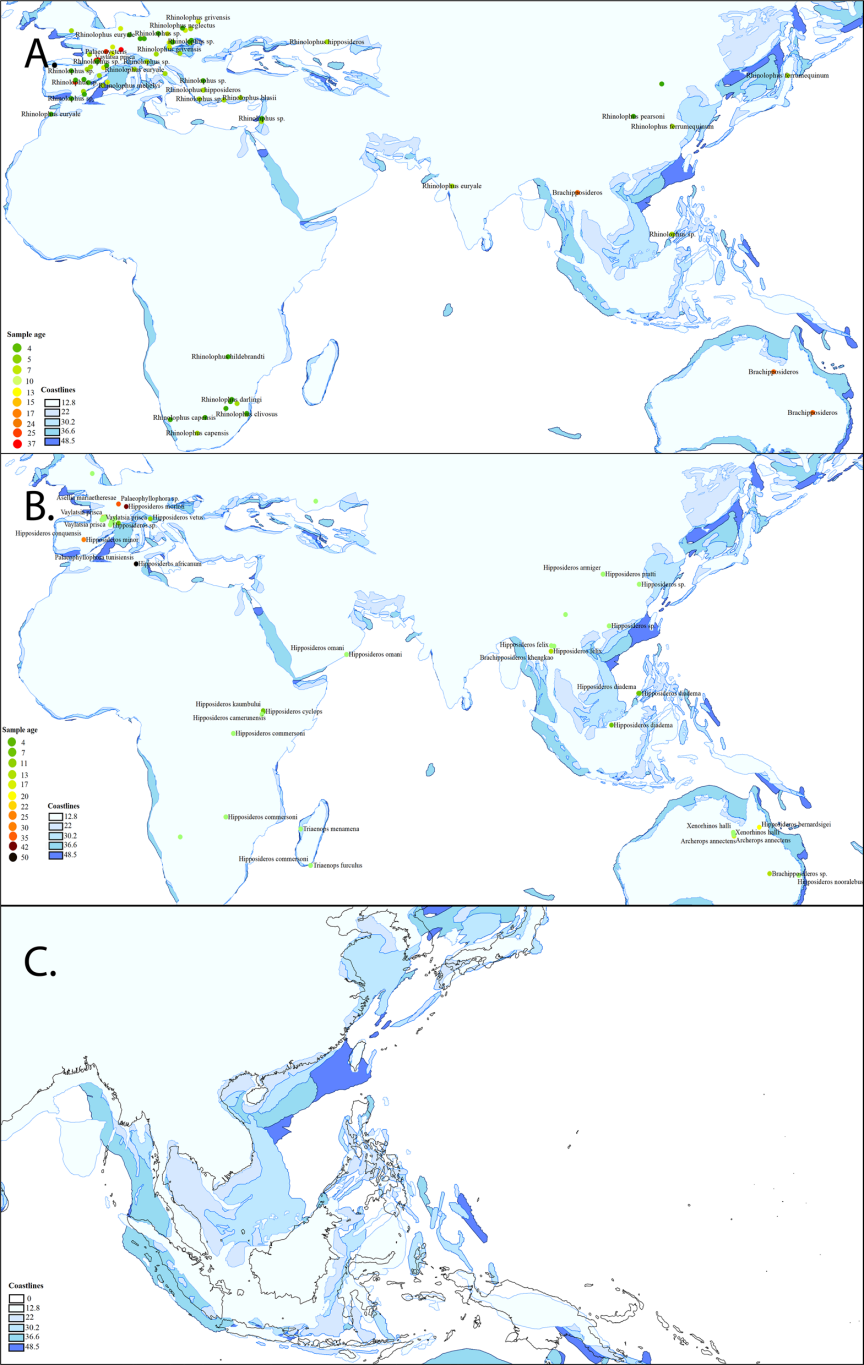
Palaeo-map and fossil occurences of Rhinolophidae. **a** Distribution of fossils Rhinolophidae in Afro-Palearctic, Oriental and Australia. Sample age and coastline in unit million of years ago, coastlines were shown per 48.5–12.8 Ma; **b** distribution of Hipposideridae fossils finding from 48.5 to 12.8 Ma; **c** changes in the coastline from the past and present forms of the Asian landmass

**Fig. 7:**
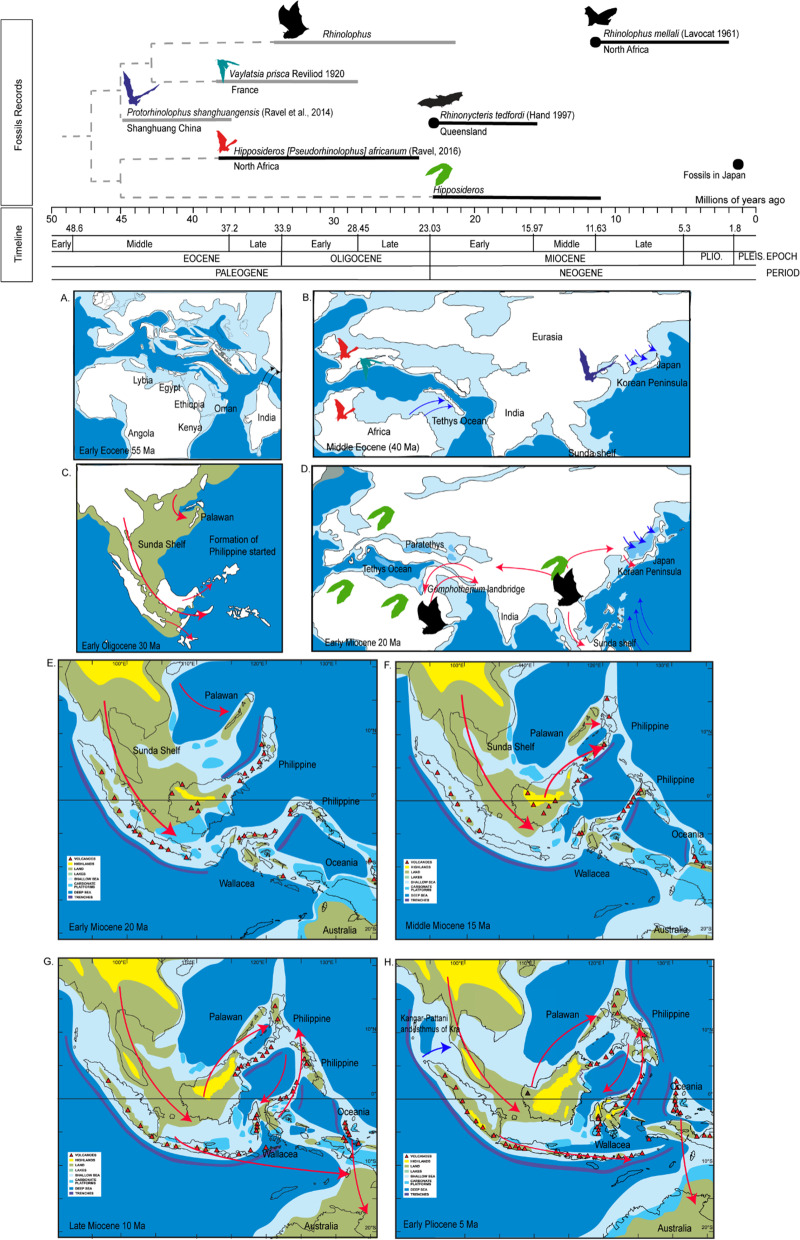
Summary of historical events in the past and main records of fossils from the common ancestor *Pseudorhinolophus, Protorhinolophus* and *Vaylatsia* in Asia and Africa (modified from Ravel et al. 2016), including of records of Rhinolophidae fossils in Australia, Japan and North Africa. Maps A–H showed paleogeographic maps redrawn from previous publications; **a** early Eocene maps showed a sea barrier between Africa and Eurasia, with India moving upward toward Asia (modified from [133]); **b** middle Eocene map indicates the appearance of ancestral Rhinolophidae and Hipposideridae (modified from [140]; **c** Early Oligocene by the initial formation of Philippine Islands and Palawan which was still connected with Asia (modified from [102]); **d** early Miocene, the closure of Tethys Ocean and formation of *Gomphoterium* landbridge (modified from [140]); **e**–**h** the Cenozoic model per five million years in Southeast Asia with the formation of Indonesia, Wallacea, Papua and the Philippines (modified from [86, 141]. The red arrows indicate possible colonization direction and blue arrows indicate the movement direction of the landmass. Bats silhoutte images were downloaded from PhyloPic (http://phylopic.org/) CC-SA

The oldest *Rhinolophus* fossils was *Protorhinolophus shanghuangensis* fossil found in Shanghuang fissures, China aged Middle Eocene [73], making it older than the *Vaylatsia prisca* (*Rhinolophus priscus*) fossil found in Europe (early Late Eocene to Early Oligocene). The oldest known fossil of *Rhinolophus priscus* is dated to the Late Eocene to Oligocene in the Quercy of France (Europe), but *Protorhinolophus* showed more primitive dental patterns, which indicates the genus is older than *Rhinolophus* [73]. The oldest fossil known in Africa, *R. mellali* of Bani Mellal in Morocco, North Africa were dated in Late Miocene to Early Pliocene, which is probably close related with *R. ferrumequinum* [30]*.* The estimated split between Rhinolophidae and its sister lineages, Hipposideridae in the middle Eocene were supported with *Hipposideros* fossils in North Africa *Pseudorhinolophus africanum* indicated the major dispersal axis of the family from North Africa to South Europe [75] (Figs. 6, 7). In contrast with Rhinolophidae, the Hipposideridae have diversified in Europe, Africa and Arabia since the middle Eocene [73], this agrees with our ancestral range estimation result, and the widespread ancestral range of Hipposideridae (Figs. 2, 6).

Another published study hypothesized that European origin of Rhinolophidae [80] but had weaker support for species in basal clades. Bogdanowicz and Owen (1992) also hypothesized that rhinolophids dispersed from Asia to Australia through Indonesia and New Guinea based on morphological data, which indicates the ancestral of *Rhinolophus* were from Asia, which is consistent with our result, and the higher rhinolophid diversity in the Asian region [6]. The colonization of *Rhinolophus* is also limited by over-water barriers, therefore limits the dispersal from Eurasia by the presence of Tethys oceans in Early Miocene (Fig. 7). In the late Oligocene and Early Miocene, the Tethys was connected the Proto-Mediterranean Sea to the Indian Ocean [81] (Fig. 7), thus may have inhibited the ancestral range expansion from Eurasia continent to Africa continent. The Afro-Arabian and Eurasian plates collided during the Middle Miocene, caused the presence of land-bridges *Gomphoterium* landbridge due to Tethys Ocean shrinkage around Late Miocene [81–84] (Fig. 7), this event may allow species to disperse from Eurasia to the Afro-Arabian continent. In general, our result suggested the ancestral Rhinolophidae originated from the Oriental region then subsequently the range expanded towards Africa through Europe, and the ancestral Hipposideridae were widespread from Afro-Arabian region to Eurasia, consistent with *Pseudorhinolophus africanum* fossils in Middle Eocene in North Africa [75].

### Ancestral biogeography range of horseshoe bats (Rhinolophidae)

Four subset analysis (Rhinolophoidea, Rhinolophidae Oriental–Oceanian–Australia lineages and Rhinolophidae-Afro-Palearctic lineage, and cryptic Rhinolophidae) suggests estimation of dispersal rate was lower than range constriction (*d* < *e*). The high *e* in best fit model demonstrated all range-changes effectively occurred anagenetically along the branches [38]. Thus, we assume the distribution of Rhinolophidae is driven primarily by dispersal (i.e., over-land range expansion, land-bridge colonization, and stepping-stone events) mixture with vicariance events. Additionally, jump-dispersal or founder-events was not well supported for Rhinolophidae ancestral ranges especially when we used bigger biogeographic ranges. However, jump-dispersal events were chosen as the best fit model for explaining oceanic-dispersal pattern when we separate each islands as different biogeographic units (i.e., Greater Sunda Island: Sumatra, Borneo and Java). Range expansion by dispersal, extinction, sympatry (subset and narrow) and vicariance events are useful to understand historical biogeographic of Rhinolophidae in Oriental–Oceania–Australia lineages, however, ancestral range of Rhinolophidae Afro-Palearctic is a better fit with dispersal, extinction, sympatry (narrow and widespread) events (without vicariance). The understanding of how organisms came to be distributed as they are also related to historical events involving complex geological history, such as glacial-inter glaciation, continental drift, biotic turnover and long-distance colonization [28]. The main event in Cenozoic era that shapes the continent including collision between the Indian plate and the Eurasian plate, created mountain ranges in Himalaya regions and acted as physical barrier for species dispersal between Indochina and India in late Eocene (around 50 Ma till present). The continuation of India attachment to Eurasia hugely affects the movement of other plates and influenced the shape of continents, archipelago and affects climatic condition in the region (Fig. 7) [46, 50, 57, 58, 85, 86].

The rapid diversification during Miocene coincided with the Mid-Miocene Climatic Optimum (around 15–18 Ma) which may provide a favourable climate in support for the evolution [87] of the *Rhinolophus* lineages. The high diversification in the Miocene not only occurred in bats, but also in the diversification of modern bird genera in Southeast Asia [61]. The basal position of two lineages in Rhinolophidae are the three species belonging to the *trifoliatus* group (*R. trifoliatus*, *R. luctus*, *R. formosae*) and *R. hipposideros.* The three *trifoliatus* species are Oriental species distributed from Indian sub-continent, Southeast Asia and Eastern Asia [5], and *R. hipposideros* is distributed throughout the Europe from Ireland in the northwest to Pakistan in the east, and south into northern regions of Africa and Saudi Arabia [88]. Phylogeographic studies suggests early colonization event of *R. hipposideros* and *R. ferrumequinum* from the east (west Asian refugium) and both of species used multiple glacial refugia across Mediterranean during the ice age [88, 89]. Last Glacial Maximum in late Pleistocene have impact on current distribution of *R. ferrumequinum*, with secondary contact was identified between Central/East China and East China/Japan [89].

Our results also show colonization of Rhinolophidae species toward Japanese archipelago may have occurred since the Middle Pliocene, which coincided with fossils from caves and fissure deposits of Middle Pleistocene, Late Pleistocene and Early Holocene in Honshu and Kyushu Island (see [90]). The colonization from continental population toward Japanese archipelago may occurred via a continuous Korean Peninsula-Japanese land bridge due to lower sea levels than at present [89], similar to the colonisation route for Asian black bear (*Ursus thibetanus*) [91].

Our result showed the colonization towards the Philippines appears to be multiple colonization events via different routes originating from the Indomalayan region (which may be via Palawan) and Wallacea region in late Miocene (Fig. 7). Similarly with Wallacean and Oceanian region, the ancestral range and colonization events in these region are complex. The thousands of islands in these regions have different continental, oceanic and volcanic origin, with many of them undergone rapid tectonic movement since Cenozoic [92, 93]. The archipelagoes (including Philippine and Indonesia) with the combination of tectonic movement, climatological oscillations and Pleistocene sea level fluctuations causing the changes of island size, connectivity and boundaries [94–96]. These important geological events may have contributed to current species distributions [97, 98], and as the effect of presence or absence of the Pleistocene land-bridge connection [95]. In addition, current distribution of species in the archipelago may be a direct result of when bats reaching the older islands longer ago and younger island more recently which leading to allopatric and vicariance speciation [99].

Two hypothesis of species biogeography in the Philippines including Pleistocene aggregate island complexes (PAICs) suggesting land exposure in the Pleistocene due to glaciation allowed species to expand their range inter five major islands [100]. Palawan Ark Hypothesis suggested species “rafted” with the North Palawan block since the separation from mainland Asia by Early Oligocene 30 Ma [50], in contact with Borneo around 15 Ma [96] and then move northward toward present position [101, 102]. The multi-route colonization of Rhinolophidae species toward the Philippines is similar to other taxa, for instance *Begonia* [103], *Cynopterus* and *Macroglossus* [104, 105]*.* This coincides with the theory of biotic colonization of Philippines, postulated as submerged land bridges, with many taxa known to have colonized Philippine through northern Philippines (from mainland Asia and Palawan) and through south route (via Sulu archipelago and Sulawesi) [106]. Previous studies also suggested that colonization of the Philippines may have taken place from the Sunda Shelf (Sumatra, Java and Borneo) and Wallacea [95].

Wallacea, including Sulawesi Island, and many small islands surrounding (i.e., Outer Banda Arc (Sumba, Timor, Babar, Yamdena, Kai, Seram), Inner Banda Arc (Bali, Lombok, Sumbawa, Flores, Alor and Wetar), Halmahera etc.) result from complex tectonic plate movement from Australian and Asian plate. Some of the Island were never connected in the past (e.g. Inner and Outer Banda Arc), and Inner Banda Arc exposed the land above sea-level and therefore permitted the colonization in Lesser Sunda Islands around 3 Ma [107]. The current arrangement of the islands provides a series of stepping stones facilitating movement of terrestrial mammals, which may include volant mammals, to colonize the Australian region [47].

Furthermore, glacial and sea-level fluctuation repeatedly formed land bridges in Pleistocene [108] and the landmass between Asia, Sumatra, Borneo and Java islands (Sundaland) [50] allowed colonialization and range expansion of Rhinolophidae from Indochina towards Sumatra and Borneo, which is in line with our results here. The diversification of *Rhinolophus* species in Borneo Island may coincide with separation of Sundaic and Indochinese rainforests [53]. The rainforest refugia in some parts of Borneo and Sumatra may allowed populations of forest-species to diverge and adapted with local climatic conditions and environment [53, 109, 110]. The glaciation and interglacial events during Plio-Pleistocene caused dramatic changes in climate, forest cover and the connection between land areas allowed species to colonized different geographic ranges. Recent diversification within species groups in Pleistocene may coincide with climatic fluctuations which affects the vegetation transition in the region, and the possible savanna corridor in part of the region [109, 111], thus indirectly influencing diversification of forest-dwelling mammals [112] and insects [113]. The divergences of *R. affinis* and *R. pusillus* lineages in Malay Peninsula during Plio-Pleistocene may coincide with major events in the peninsula. The possible flooding of Isthmus of Kra during Pliocene, and persistent climatic transition in this region [110], the adaptation to specific climatic conditions and long term ecological differences, combined with the peninsula effect may cause the faunal transition between Indochinese taxa and Sundaic taxa, with major transitions at the Isthmus of Kra, and the Kangar Pattani line [56, 110, 112, 114] (Fig. 7). Furthermore, the sea-level rises during Pliocene isolated Sumatra, Borneo and Java Islands in Indonesia created physical barriers between Indochina and species from the islands [50, 61]. Borneo is the largest landmass of former Sundaland and has served as stable land for at least 20 million years and was less affected by sea-levels changes compared with Sumatra (which has come together as a stable from 5 to 10 Ma) and Java (2–5 Ma) [53, 61].

High rhinolophid diversity in Asia compared to the other biogeographic regions may be expected as many species are restricted to islands or group of islands, for instance four endemic species in Japan (i.e., *R. cornutus*, *R. pumilus*, *R. perditus*, *R.imaizumii*) (Ohdachi et al. 2015) and at least four endemic to the Philippines (i.e., *R. inops, R. rufus, R. subrufus, R. virgo*) and various species endemic in Indonesia islands (i.e., *R. nereis, R. madurensis, R, keyensis, R. montanus, R. euryotis, R. celebensis, R. canuti* [115], which represents physical barriers for bat dispersal such as water and mountain ranges. In contrast, Palearctic regions were influenced with repeated glaciations and in Africa, that may cause high species turn-over and high rates of gene flow which decelerating speciation compared to complex biogeographic areas in Asia [74]. The dispersal of Rhinolophidae species to Africa may be via forest corridors that appeared during middle Eocene because of the warm climate [116], and the dispersal of ancient species throughout the southern Palearctic and Mediterranean, through Sahara Arabian and eventually into Africa [18]. The basal lineages within African radiation are *R*. *landeri* and *R. alcyone*, which occur in rainforests and may indicate early colonization around the late Oligocene (~ 20 Ma) through forest affinities as predicted in [20]. The closure of the Tethys Ocean us estimated around the Oligocene (around 27) Ma to Miocene [81, 117, 118]. A land bridge in Arabian Peninsula which connected Asia and Africa, and may have facilitated dispersal of many animals lineages in the Miocene such as lizards [119], frogs [120], chameleons [121], and butterflies [122, 123]. As the consequences of shrinkage of Tethys sea, desert and arid conditions expanded across North Africa in the Late Miocene (around 7 Ma), marking the origin of Sahara Desert and the Middle East Desert and the Arabian Peninsula [82]. Arid adapted species colonized Africa and now inhabit most of savanna region in Sudanian, Somalia, Ethiopia and deciduous woodland in Southern Africa [21, 30].

Unlike Hipposideridae, there are currently no records of Rhinolophidae species in Madagascar. Madagascar + India + Africa are ancient fragments of Gondwana and has been separated from Gondwana since 120 Ma and, and Madagascar separated from India by 90 Ma [124]. Madagascar started to break away from Africa around 165 Ma [125] and to become isolated in Cretaceous [126], and the invasion from Africa continent toward the island may not occurred due to broad watergaps as physical barriers for dispersal as Rhinolophidae are weak fliers [30]. Additionally, higher diversity richness and endemism in Madagascar appears as a result of dispersal from Africa and followed with diversification [127, 128], and typically reflects more recent events around Plio-Pleistocene [129].

The *d* and *e* parameters may show different rates depending on the bats family, for instance in Pteropodidae [130]. We assume the historical biogeography in bats varied between families which may relates to flight performance between species and differences in dispersal ability. For instance bat colonization to Madagascar includes most bat families (Pteropodidae, Emballonuridae, Hipposideridae, Vespertillionidae, Nycteridae, Molossidae and Myzopodidae), with a notable of absence Rhinolophidae species (Racey et al. 2009). Wing-loading, wing-span, aspect ratio and wing shape are the main aerodynamic variables in determining the flight performance and flight efficiency of species. Rhinolophids bats generally possess broad and short, low wing loading and aspect ratio adapted for good maneuverability in foraging as slow aerial hawkers, perch hunters and gleaners (Norberg and Rayner 1987; Amador et al. 2020). However, flight would be expensive for a long distance flight and therefore limiting the ability for dispersal. Contrary to Rhinolophidae, most Pteropodidae possess high wing loading and large wing spans, with low aspect ratios that support for long distance travel but are less maneuverable [3], as a consequence many Pteropodids are restricted to various oceanic islands where they are often the only native mammal species.

True-flight abilities together with echolocation have been long considered as remarkable evolutionary features which have driven the success of bat species and enabled them to occupy wide range of ecological niches, but the morphological features have evolved into current form from a common ancestor [2]. Other factors such as dispersal filters (isolation, geologic history), environmental filters (present and past climate, and environmental heterogeneity) have also influenced the diversity of vascular plants [131] and may also have contributed in shaping the current biogeographic ranges of Rhinolophidae.

### The evolutionary history of species divergence in potential cryptic Rhinolophidae and the comparisons with previous study

Indomalaya is a region with particularly high species diversity of Rhinolophidae and Hipposideridae. However, many species are cryptic, suggesting the number of species currently underestimated [70]. Around 40% of rhinolophids species are potentially cryptic based on integrative taxonomic approaches, with estimated around 44 potential cryptic species from total of 10 *Rhinolophus* species sensu lato [6]*.* However, a comparison of time divergence estimation across the genes is challenging because of the limited number of species used in previous studies, and the paucity of the bat fossil record. We acknowledge the disconcordance of time tree in this study with other studies, which was also due to differences in systematic taxonomic coverage and genes being used (as described in the “Introduction” section). Although we found some discordance between tree topologies we generated in this study and from previous studies, we attained similar results across subset analysis related to ancestral range estimation in Rhinolophidae. In terms of species coverage from South China and the Southeast Asian region, we covered most described species from each geographical region. Our results suggest current species sensu lato started to diverge in the late Oligocene and Miocene, meanwhile the potential cryptic species in the region diverged in the Plio-Pleistocene epoch. This analysis indicates that geological events during the epoch contributed in shaping current cryptic diversity patterns seen today. Future directions should aim to include all Rhinolophoid species distributed in the Old-World tropics, as we currently cover around 108 (52%) species of total species identified to date (210 species; Rhinolophidae = 110, Hipposideridae = 90, Rhinonycteridae = 9 species) (Simmons and Cirranello, 2021).

### The systematic and ancestral biogeography range of Hipposideridae and Rhinonycteridae

Hipposideridae and Rhinonycteridae are sister families within Rhinolophoidea superfamily. Amador et al. (2018), suggested the phylogenetic tree for superfamily as (Rhinonycteridae, (Hipposideridae, Rhinolophidae)) but Foley et al. (2015) resolved the relationship as (Rhinolophidae, (Hipposideridae, Rhinonycteridae)), similar with species tree in this study [43]. Foley et al. (2015) suggested the African species of *H. abae*, *H. caffer* and *H. jonesi* were within Asian *Hipposideros*, and *Coelops frithii*, *Aselliscus stoliczkanus* were sister to *Hipposideros* African species (*H. commersoni* and *H. vittatus*). Nevertheless, this arrangement has low branch support, and low taxonomic coverage which only includes total of 13 species of Hipposideridae Asian lineages and Afrotropical lineages. In the species tree use in this study, [43] resolved the relationship and *H. jonesi* (Afrotropical species) species fall within Oriental–Australian lineages, similar with tree topology of Amador et al. (2018), paraphyly with *Aselliscus* sp., *Coelops frithii* and *Anthops ornatus* in the same lineage, which is in agreement with [132]. *H. abae* and *H. caffer* as Afrotropical species are within Afrotropical lineages (in contrast with Foley et al. 2015, but in concordance with [60]) with *Asellia tridens* in the basal of Afrotropical and Oriental–Australian tree.

For Rhinonycteridae, here we include seven species out of nine species within the family, belonging to four genera, *Triaenops*, *Paratriaenops*, *Cloeotis*, distributed in Madagascar and Afrotropical, and *Rhinonycteris* endemic to Australia with tree topology as [(*Cloeotis*, *Paratriaenops*), (*Rhinonycteris*, *Triaenops*)]. This arrangement also in contrasts with Foley et al. (2015) with tree topology as [(*Paratriaenops*, ((*Triaenops*, *Cloeotis*), *Rhinonycteris*)] while using six species, and Amador et al. (2018) [*Paratriaenops*, *(Triaenops*, *Cloeotis*)] with absence of genus *Rhinonycteris.* Certainly, missing taxa and gene choice compromise these results to at least some degree, thus the tree generated in Álvarez-Carretero et al. 2021 using supermatrix genome-scale of 182 genes may assume as the latest update tree with smaller uncertainties which facilitates precise testing of historical biogeographic analysis.

The historical biogeography of family Hipposideridae is also related to complex historical geology of the Old-World regions (see discussion above) (Fig. 7). The ancestral ranges of Hipposideridae suggests an Oriental and African origin, the Oriental–Oceania–Australia lineages supports the jump-dispersal for species colonization. This explains how new lineages colonized between regions (such as island), and allowed inter-continental disjunction or oceanic-dispersal pattern (dispersal without range expansion) (Matzke 2014).

In contrast with Rhinolophidae, their dispersal rate is higher than range constriction in Hipposideridae. This indicates that ancestral Hipposideridae were able to disperse across water-bodies or used land-bridges and stepping stones. The colonization of Hipposideridae towards Greater Sunda Island may have occurred when the land was connected with Peninsula since early Miocene, followed with multiple fluctuation of sea level until the Sunda Island formed separately at 5 Ma [50, 57, 58, 86], similarly with Rhinolophidae. The Wallacea region might have played an important role as a stepping stone to colonize Oceania and Australia regions, but migration, population exchanges and secondary contact may have occurred in the past between Oceanian-Wallacean region. In concordance with Rhinolophidae ancestral ranges, the range expansion of Hipposideridae to Africa may also occurs via Arabian Peninsula and Sahara-Arabian around Oligocene periods (27–30 Ma). Even though the *Gomphoterium* landbridge and Tethys Ocean closed around the Late Miocene, the marine barriers did not totally prevent mammalia exchanges between Eurasia and Afro-Arabian, for example for proboscideans [133]. Ancestral Hipposideridae were widespread in Eurasia and Africa since the end of Early Eocene with the evidence of fossils records in Africa and Arabia [75].

The colonization of Hipposideridae towards the Philippines, Wallacea, Oceania and Australia coincided with acceleration of the orogeny of Philippine archipelago, Wallacea with the orogeny of Sulawesi and the main stages of the New Guinean orogeny in Oligocene [50, 86, 122]. Our result suggests Oceanian colonization since the early Miocene (~ 20 Ma), similarly with passerine birds by [134]*.* However, central range of present day of Papua New-Guinea likely did not begin to appear as land until the early-middle Miocene (14–16 Ma) [135] and the present form is predicted since 4–5 Ma. Therefore, founder-events may have involved in island-hopping across the final fragments of a proto-Papuan archipelago in Hipposideridae [136].

Rhinonycteridae split earlier from the common ancestor Hipposideridae Afrotropical lineage around Middle Miocene 40.55 Ma (95% HPD = 36.98–44.14 Ma), but species diversification occurred since 21.88 Ma (95% HPD = 16.54–28.48 Ma) with most extant species distributed in Madagascar, except *Rhinonycteris aurantia* which is distributed in Australia. Our result suggests the origin of Rhinonycteridae in Madagascar was Sudanian + Somalia + Ethiopia with *Paratriaenops* and *Triaenops* was recently emerged in Plio-Pleistocene epoch (1–3 Ma). This coincided with previous studies, which stated that most the present-day organism in Madagascar are of predominantly African origin [127]. Dispersal over-water may explained the colonization of ancient Rhinonycteridae in Madagascar, followed with diversification and in-situ radiation, which coincided with Plio-Pleistocene climate cycles [129]. The ancestral *Cloeotis percivali* may have high dispersal ability allowed the ancestor to exchange from Madagascar to Southern Africa. This associated with our result that suggests higher extinction rate in these lineages, indicating that the extinction of species in the ancestral range was followed by the colonization of descendants to the new area. The endemic species in Australia belongs to Rhinonycteridae, *Rhinonycteris aurantia* is the only species that currently distributed outside Africa continent [15]. The colonization event of this species towards Australia is challenging to explain, our result shows an African origin but it is almost impossible to explain range extension from Africa to Australia with diversification around Miocene. Some possible hypothesis maybe long distance dispersal over water barrier from Africa to Australia, however the dispersal mechanisms are unknown considering the species are weak fliers. Some of hypotheses suggest that bats colonized Australia by storm-blown to continental shore, for example red flying foxes (*Pteropus scapulatus*) [137], though landbridges between North Australia and Papua existed for extended periods of time. Ancestral *Rhinonycteris* may have entered Australia before the Miocene, with fossil evidence of *Rhinonycteris tedfordi* dating around Miocene from Riversleigh, Northwestern Queensland [138]. Alternative hypothesis suggests waif dispersal and stepping stones through Asia, Sundaland, Wallacea and Oceania [137], therefore, how this species colonized the region still debatable, but low diversity means that species in intermediate regions may have become extinct.

Finally, as we stated in the results above, the inferences of early Hipposideridae ancestral ranges are weakly supported for each state. This issue may can be alleviated if the Hipposideridae are analyzed as a whole, however some extant species were widespread (i.e., *H. ater, H. cervinus, H. galeritus, H. diadema*) in 6–7 zoogeographic regions (India, Indomalayan, Greater Sunda Islands, Philippines, Wallacea, Oceania and some in Australia, though further analysis may reveal undescribed species in these groups), causing large computational demands to analyze whole family. Reducing number of zoogeographic regions would facilitate processing, but would give the similar results as ancestral range reconstructions presented in Fig. 2. In addition, further systematic research needed for these widespread species that may contain undetected cryptic diversity [139]. For instance, there are multiple potential species listed as *H. ater* within Southeast Asia and Australia [132]. Resolving the systematic and taxonomic issues, which if resolved would strengthen the inferences of ancestral range estimation within this family.

## Conclusion

The results presented here provide an overview of biogeographic history of extant Rhinolophoidea superfamily (Rhinolophidae, Hipposideridae and Rhinonycteridae). The result suggests that the Oriental origin, and the Indomalayan is the ancestral origin of Rhinolophidae and Hipposideridae, but Rhinonycteridae ancestors originated in Africa. The result showed a lower dispersal rate (range expansion) than extinction rate (range constriction) in Rhinolophidae, but in contrasts with Hipposideridae Oriental–Oceania–Australia lineage. Jump dispersal events explain the ancestral range in Hipposideridae Oriental lineages. Current distribution of extant taxa appears to be a result of a combination of dispersal, extinction, sympatry and vicariance events, followed with complex geological history of the Old-World regions. Further study combining complete species coverage of Rhinolophidae, Hipposideridae and Rhinonycteridae may increase the resolution of the historical biogeography of these taxa.

## Methods

### Sampling, systematic coverage and biogeographic sampling

In total we included 104 species of Rhinolophidae (n = 47; 43% from 110 total described species), Hipposideridae (n = 50; 56% of 90 species) and Rhinonycteridae (n = 7; 78% of 9 species) in this study. Taxonomic sampling coverage was based on number of species used in the tree published in Álvarez-Carretero et al. 2021. The total species coverage per biogeographic area was; Australia–Oriental–Oceania (thereafter, AOO) = 31; Afrotropical–Madagascar–Palearctic (thereafter AMP) = 16 for Rhinolophidae and AOO = 38; AMP = 12 for Hipposideridae. The term of AMP is used for all subset analysis for consistency, but it is important to note there is no record of Rhinolophidae species in Madagascar to date (see details in “Discussion”). In addition, total of 26 Rhinolophidae sensu lato mainly distributed in Southeast Asia and their potential cryptic species (44 potential cryptic [6]) was used to assess their evolutionary history and ancestral biogeographic range.

### Biogeographic mapping

To assess species routes based on fossil data and assess the correspondence between fossil data (where present) and ancient coastlines we downloaded recent files showing coastlines for periods between the present (0 Ma) and 45 Ma, including 37 Ma, 30 Ma, 22 Ma, 13 Ma based on [40]. Fossil data from Rhinolophids was then downloaded from the Fossilworks (http://www.fossilworks.org/cgi-bin/bridge.pl?a=taxonInfo&taxon_no=40644) and Global Biodiversity Information Facility (GBIF), using a criteria of fossil species and the search term Rhinolophidae (https://doi.org/10.15468/dl.uy9gx5). This was repeated for Hipposideridae (https://doi.org/10.15468/dl.2826pj). Data was cleaned to give putative species name at the highest available level, and age of fossil in millions of years, dates were also checked from associated publications when not available in the spreadsheets. This data was rounded up to the nearest million years (due to uncertainty and ease of analysis and display), and where not available for any given fossil the dates for the same species in other parts of the region was used where present for mapped ranges of species.

### Biogeographic analysis of old-World Rhinolophidae and Hipposideridae

We performed ancestral geographic range analyses using probabilistic modelling in R package “BioGeoBEARS version 1.1.2” [39]. We statistically compared the likelihood-based model of geographic range evolution of DEC model (Dispersal–Extinction–Cladogenesis) of LAGRANGE [41], a likelihood implementation of the processes assumed by parsimony of DIVA [42] (therefore named DIVALIKE), a likelihood version of the range evolution model of BAYAREA [37] (therefore named BAYAREALIKE) and a modification of DEC model by prohibiting the transition into null-range (DEC*) [38]. Each model is fully parameterized in BioGeoBEARS supermodels with different assumptions about anagenetic and cladogenetic change processes. A free parameter of “*j*” (jump dispersal or founder-event speciation) was added in DEC + J, DEC* + J, DIVALIKE + J, BAYAREALIKE + J models and nested with another two free parameters (*d* and *e*) within DEC + J, DEC* is nested within DEC* + J, DIVALIKE is nested within DIVALIKE + J and BAYAREALIKE is nested within BAYAREALIKE + J [39].

To infer the ancestral biogeographic ranges of Rhinolophidae and Hipposideridae, we used a dated-tree generated from [43] which used a supermatrix of 33.2 × 10^6^ alignment length from 72 genomes (15,268 genes). The date estimation in this published study produce smaller uncertainties in dates estimates, thus are recommended for macro-evolutionary studies [43], including ancestral biogeography (Additional file 1: Fig. S1). There are some limitations in statistical analyses of ancestral range evolution. The 14 areas coded here would produce a huge transition matrix (2^14^ = 16,384 possible area combination; 16,384 × 16,384 transition matrix (268,435,456 total) that has to exponentiated across each tree branch for maximum likelihood optimization. This large matrix is currently not feasible with matrix-handling algorithms. Therefore, here we analyzed the ancestral ranges in 4 subset analyses, including: (1) global Rhinolophoidea superfamily (Rhinolophidae, Hipposideridae and Rhynonycteridae), we then subdivided the analysis into sub-regions for each family into: (2) Rhinolophidae Asia–Australia lineages and Rhinolophidae Afrotropical–Palearctic lineages, (3) Hipposideridae Asia–Australia lineages and Hipposideridae–Rhinonycteridae Afrotropical lineages, (4) Potential cryptic species of Rhinolophidae in Asia lineages (potential cryptic species were delineated based on integrative taxonomic approaches as reviewed in [6]). This analysis strategy was to optimize the computational efficiency by considering the possible numbers of states for each sub-analysis. The input files for analyses required a dated-tree and geography file. We pruned the species tree published from Álvarez-Carretero et al. 2021 to Yinpterochiroptera lineages and trimmed the branches to Hipposideridae, Rhinonycteridae, Rhinolophidae using the function drop.tip in R package ‘phytools’ v1.0-1 [44], and removed the outgroups. We merged the subspecies in the tree tip into species level (OTUs) with taxonomy following Simmons and Cirranello 2021, using R package BioGeoBEARS v1.1.2 [39] in the function prune_specimens_to_species. The geography files are PHYLIP-formatted files, and were also used for C++ LAGRANGE editor file, which was generated in R using function save_tipranges_to_lagrangePHYLIP. To determine the biogeographic region, we used the current distribution of extant taxa from the data provided in previous literature and combined this with data from the Global Biodiversity Information Facility GBIF (https://www.gbif.org/) (https://doi.org/10.15468/39omei), as IUCN ranges of bats may be unrepresentative and risk being particularly inaccurate [45]. We mapped the current occurences record of each species and codes area based on the distribution in each zoogeographic zones. The division of zoogeographic zones were defined based on geologic history of the zones and areas of endemism which was defined based on previous studies [26, 46–56] (Fig. 1, modified from Holt et al. 2013).

### Ancestral biogeography ranges estimation of Rhinolophoidea superfamily

For biogeographic regions of Rhinolophoidea superfamily, we divided the current known species distribution into nine zoogeographic areas using an updated version of Wallace’s zoogeographic regions of the world constructed on the distribution and phylogenetic relationship on 21,037 species of amphibians, birds and mammals [55]. The regions are shown in Figs. 1 and 5: Afrotropical + Madagascar (A); Oceanian (B); Philippine (C); Sino-Japanese (D); Oriental (including India + Indo-Malaya (Southeast Asia + Greater Sunda Islands (Sumatra, Java, Borneo)) (E); Palearctic (Europe, Mediterranean) (F); Sahara-Arabian (G); Australia (H); and Wallacea (Sulawesi, Lesser Sunda Islands and area in vicinity) (I). We assessed the biogeography of Rhinolophoidea across the tree, with 104 species at the tips (outgroup removed). We set the maximum area range to five (according to number of maximum areas occupied by extant taxa) resulting in 382 and 381 possible ranges with and without null-range, respectively**.**

### Ancestral biogeography ranges estimation of Rhinolophidae family

Secondly, we pruned the *Rhinolophus* species from the tree of Asia lineages, consisting of 27 species sensu lato. The pruned tree did not include three species of *R. hipposideros*, *R. formosae*, *R. luctus* which are diverged early in the Oriental and Afrotropical lineages in the initial trees (Fig. 5). We attempted to prune Rhinolophidae as a whole family including all lineages. We included 47 species (OTUs) distributed in 13 biogeographic areas and 5 areas maximum occupied with extant taxa resulting in 2380 of possible geographic ranges. This caused computational issues, thus analysis was run separately for each lineage. We divided the analysis into *Rhinolophus* of AOO and the AMP subdivision. For the AOO clade, we divided the biogeographic regions into a smaller divisions (as shown in Fig. 2a) consisting of: Sino-Japanese (A); India (B); Indomalayan (C); Greater Sunda (Sumatra + Java + Borneo) (D); Philippine (E); Wallacea (Sulawesi + the Lesser Sunda Islands + Halmahera (from Wallace line to Lydekker line); Oceania (G); and Australia (H) [46, 47, 53, 55, 57, 58]. The total maximum area range was set to five, based on the number of areas occupied by extant species, (i.e. *R. affinis* with current distribution in India, Indomalaya, Greater Sunda, Philippines, and Wallacea), resulting in a total of 219 areas occupied.

In addition, we pruned Rhinolophidae in the AMP region consisting of 16 species, and number of maximum areas occupied by one species were set to 4, resulting 57 possible geographic ranges. The geographic areas included: Sahara-Arabian (A); Sudanian + Somalia + Ethiopia (B); Guinean + Congolian (C); Zambezian + Kalahari + Southearn Africa (D); Europe and Mediterranean (E); and Sino-Japanese (F) following biogeographic division of Africa, Palearctic and Sino-Japanese (as shown in Fig. 2b) [55, 59].

### Ancestral biogeography ranges estimation of Hipposideridae and Rhinonycteridae family

We first ran Hipposideridae and Rhinonycteridae analysis across the entire old-world biogeographic region from 13 total biogeographic regions, of 57 species (OTUs) and seven areas that can be occupied by a single species. This calculated the total number of possible geographic ranges of 5812 states and created a large matrix (33,779,344 total) that could not be handled with current hardware and matrix-handling algorithms. Thus, we analyzed the AOO lineages separately from AMP lineages. For Hipposideridae AOO lineages, total of 39 species distributed in nine biogeographic regions were included in the analysis and a species can occupied a maximum of seven biogeographic areas, resulting in 502 possible geographic ranges. The biogeographic areas are shown in Fig. 3a including: Sudanian + Somalia + Ethiopia (A); Sino-Japanese (B); India (C); Indomalayan (D); Greater Sunda islands (Sumatra + Java + Borneo) (E); Philippine (F), Wallacean (G); Oceanian (H); and Australia (I). The biogeographic region of Sudanian + Somalia + Ethiopia (A) was being included in the Asian–Australian clades as *H. jonesi* appears to be sister to the Asian clades, and is likely to reflect an earlier African–Asian colonization event [60].

We pruned the AMP branches of Hipposideridae and the sister family Rhinonycteridae [14, 15]. The total number of species for this subset analysis is 18 distributed in six biogeographic regions, and five total area occupied by single extant species (numbers of possible ranges = 63). The biogeographic areas are shown in Fig. 3b including: Sahara-Arabian (A); Sudanian + Somalia + Ethiopia (B); Guinean + Congolian (C); Zambezian + Kalahari + Southern Africa (D), Madagascar (E); and Australia (F).

### Ancestral biogeography ranges estimation of Cryptic Rhinolophidae in Asia

Additionally, we use the maximum clade credibility (MCC) tree from BEAST analysis (see below) to estimate the ancestral range of potential cryptic rhinolophids species in Asia (reviewed and delineated in [6]). In this subset analyses, we seek further detail of how the ancestors of recently diverged lineages expanded and contracted. The biogeographic units were split into eight “areas of endemism” of Southeast Asia and the West Pacific [47, 54, 61] including India (I), Himalaya (H); Southeast Asia (S); China (C); Isthmus of Kra region to Kangar-Pattani (K); and Kangar-Pattani line to the tip of Malay Peninsula (P); Borneo (B); and Sumatra (S) (as shown in Fig. 4). Maximum range size in analysis were set to eight (to match the number of regions) based on the assumption that OTUs (species) could occur in all areas. These areas represent the biogeographic zones used in former biogeographic analyses [47, 54, 61]. Area assignments for each OTU were based on current species distribution known from sequenced individuals which were cross-referenced with each biogeographic region they were recorded in ArcMap 10.3 to determine the range each OTU occurred in. The OTUs as tip nodes represent the species and cryptic species assigned from phylogenetic tree based on Maximum Likelihood and Bayesian Inferences.

For all above analyses, we use R package GenSA [62] (Generalized Simulated Annealing) to optimize the maximum likelihood calculation for all models in BioGeoBears. All BioGeoBEARS supermodels run under non-time-stratified analysis. We use statistical model comparison to compare the best-fit model given the data, a likelihood ratio test (LRT) for nested models and Akaike Information Criterion corrected (AICc) and weighted (AICw) were used for the non-nested models to observe the best model among all biogeographic scenarios.

### Evolutionary history of potential cryptic Rhinolophidae species in Asia

To assess the evolutionary relationship and to estimate the time divergences between potential cryptic species, we ran BEAST v2.6.3 [63]. Potentially cryptic species in the region were delineated using integrative taxonomic approaches by combined phenotypic, acoustic and genetic data (as detailed in Chornelia et al. 2022) [6]. The tree was constructed based on 26 Rhinolophidae species senso lato distributed in Southeast Asia and India, using mtDNA COI 680 bp. The sequences were acquired from this study and mined from GenBank databases and from our previous work in the region (GenBank accession number: OK483366; OK483495–OK483509;OK562850–OK563036; OK563109; and OK563727) (see Chornelia et al. 2022 for all details of samples). Inferring evolutionary history using a single genes can be problematic for a variety of reasons, nonetheless, this is the available data in this region with higher systematic coverage [6]. Therefore, a care is needed in inferring the result and broad comparison with previous studies is crucial to provide an accurate understanding of species shifting ranges. Alignments were conducted in MAFFT using G-INS-i strategies, the output (in FASTA file) then converted into NEXUS file using Mesquite v3.6 [64], prior setting in.xml file was generated in BEAUti v2.6.3 [63] and SSM (Standard Substitution Models) package was loaded in BEAUti prior the analysis,. Relaxed clock log normal [65] was used to allow the clock rate to vary across the tree branches. We used a Birth–Death prior in the tree model which provided better accuracy and provides more precise result in all speciation scenarios based on previous studies [66]. The secondary calibration dates were taken from published papers due to the scarcity of bat fossils. Here we; (1) estimated the time divergence between Rhinolophidae and Hipposideridae was between 39 and 45 Ma, based on previous estimates including 41 Ma (95% highest posterior density (HPD) = 37–47 Ma) [22]; 39 Ma (95% HPD = 37–43 Ma) [67]; 42 Ma (95% HPD = 39–45 Ma) [14]. (2) To estimate time divergence between closely related species, we use Foley et al. (2015) for node calibration between *R. shameli* and *R. creaghi* (4 Ma (95% HPD = 3–5 Ma); *R. trifoliatus-R. luctus* (3 Ma (95% HPD = 2–4 Ma) and in addition, we calibrated node between *H. armiger* and *A. stoliczkanus* (31 Ma (26.5–31.5 Ma)). Thus, we set the priors to calibrate the nodes in BEAUti with parameterization as follows: (1) For Rhinolophidae and Hipposideridae, we selected *H. armiger* and *R. sedulus,* with prior distribution means (M) = 42 and standard deviations (S) = 0.045 which specifies that the distribution of priors is centred at 42 Ma and 95% probability range covering at 39–45 Ma; (2) *R. shameli* and *R. creaghi* prior, M = 4, S = 0.15 (median = 3.92 Ma. 95% probability range = 3.09–5.06 Ma); (3) *R. trifoliatus* and *R. luctus* prior, M = 3, S = 0.23 (median = 2.92 Ma, 95% probability range 2–4.27 Ma); (4) *H. armiger* and *A. stoliczkanus* prior, M = 30, S = 0.6 (median = 29.9 Ma, 95% probability range 27.1–33.1 Ma). All calibration priors are set to log-normal, each configuration of 2.5% quantile and 97.5% quantile set in ‘mean in real space’ and none of the prior settings enforce to be monophyletic as each “species” consist of several clades (as many of these are species complexes). MCMC algorithms were set to run for 50,000,000 cycles, trees stored every 1000 cycles. The trace files generated from BEAST were analyzed in TRACER1.7 [68]. 10% of initial tree were discarded in TreeAnnotator v2.6.3 [63]. The final tree was visualized in FigTree and finalized the graphics in Adobe Illustrator. The maximum clade credibility tree from the output was used for BioGeoBEARS analysis.

## Supplementary Information

**Additional file 1.** Supplementary figures.

## Data Availability

All data used in this study are provided in this document.

## Abbreviations

AOO: Australia–Oriental–Oceanian
AMP: Afrotropical–Madagascar–Palearctic
BioGeoBEARS: Biogeography with Bayesian (and likelihood) evolutionary analysis in R script
Ma: Million of years ago
GBIF: Global Biodiversity Information Facility
DEC: Dispersal–extinction cladogenetic
*d*: Dispersal
*e*: Extinction
*j*: Jump-dispersal or founder-event
BEAST: Bayesian Evolutionary Analysis by Sampling Trees
GenSA: Generalized simulated annealing
LRT: Likelihood Ratio Test
AICc, AICc_wt: Akaike Informatio Criterion corrected and weighted
HPD: Highest posterior density
