# Supplementary material for: The evolutionary history and ancestral biogeographic range estimation of old-world Rhinolophidae and Hipposideridae (Chiroptera)
Source: BMC Ecol Evol. 2022 Oct 3;22:112. doi: 10.1186/s12862-022-02066-x (PMC9528145; doi:10.1186/s12862-022-02066-x)

## **Supplementary Figure S1**


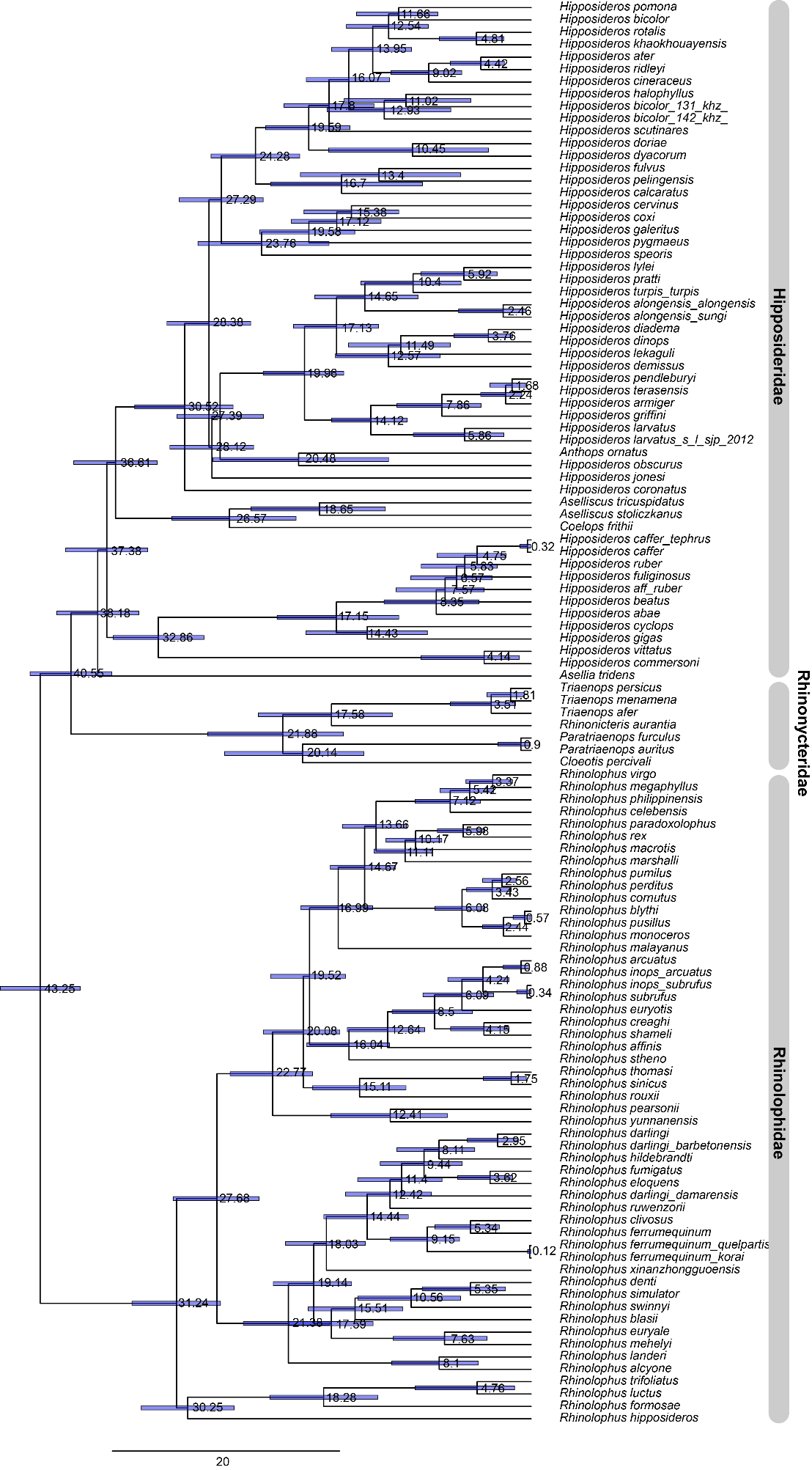


## **Supplementary Figure S2**
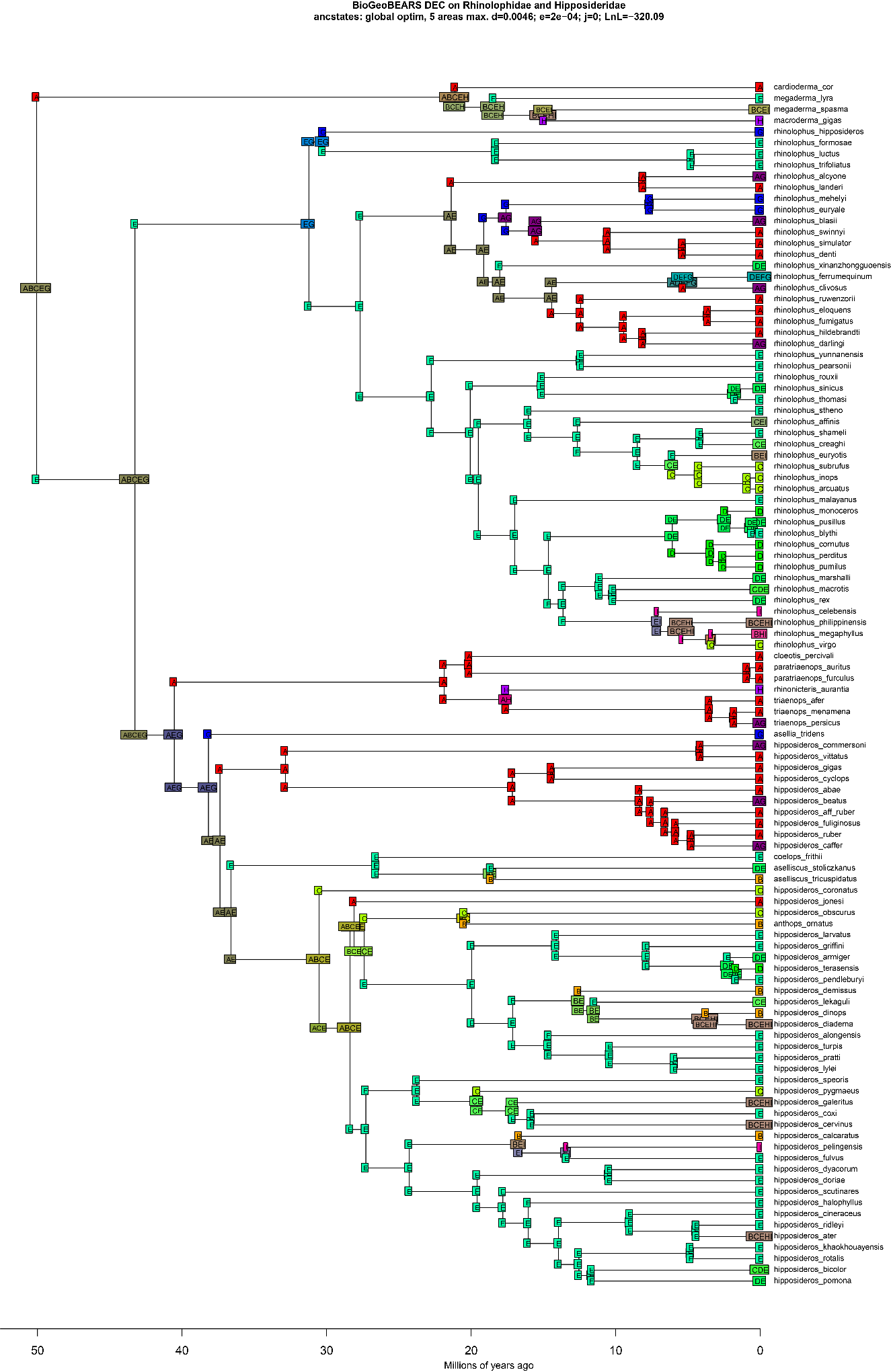


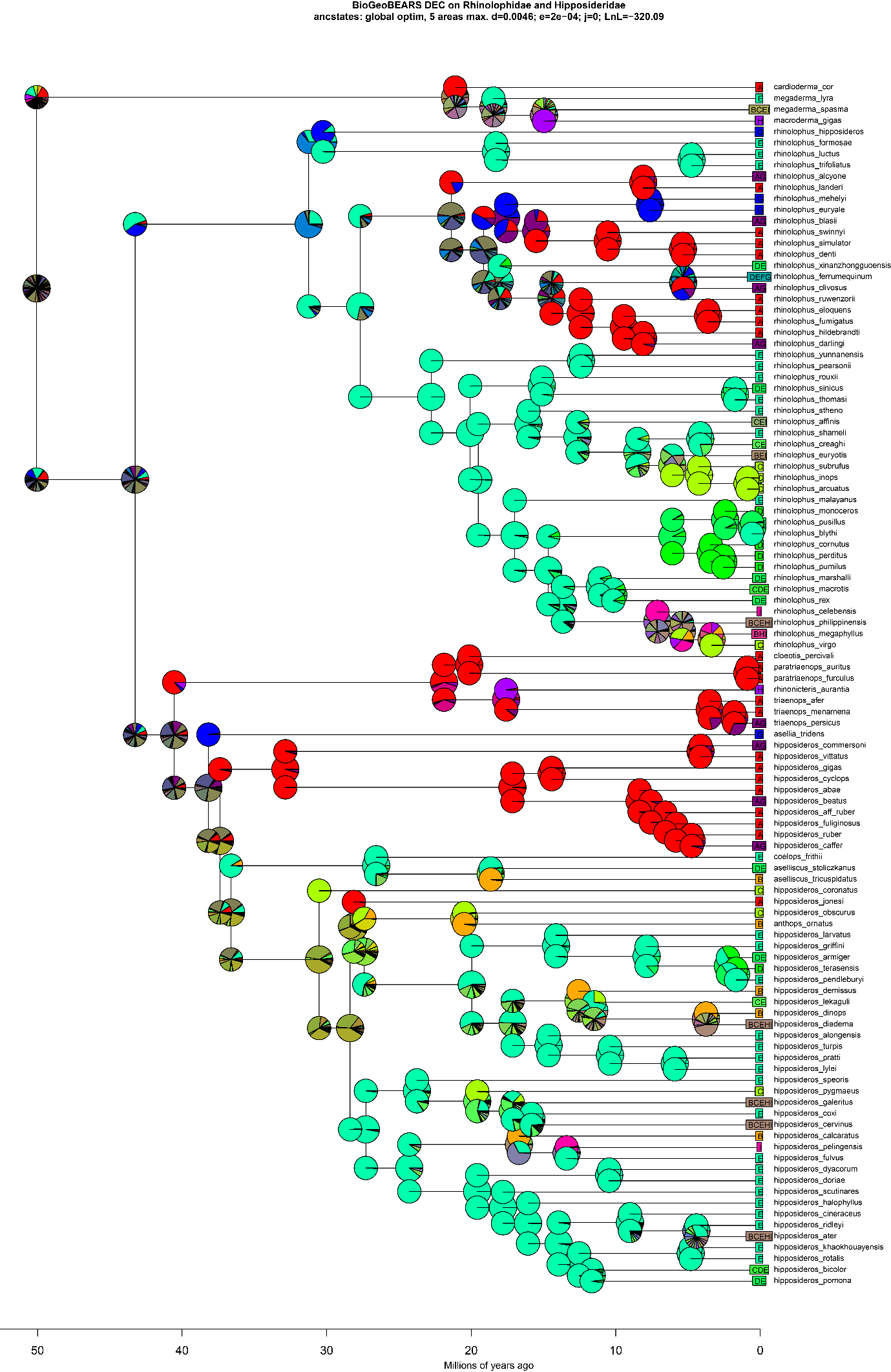


## Supplementary **Figure S3**


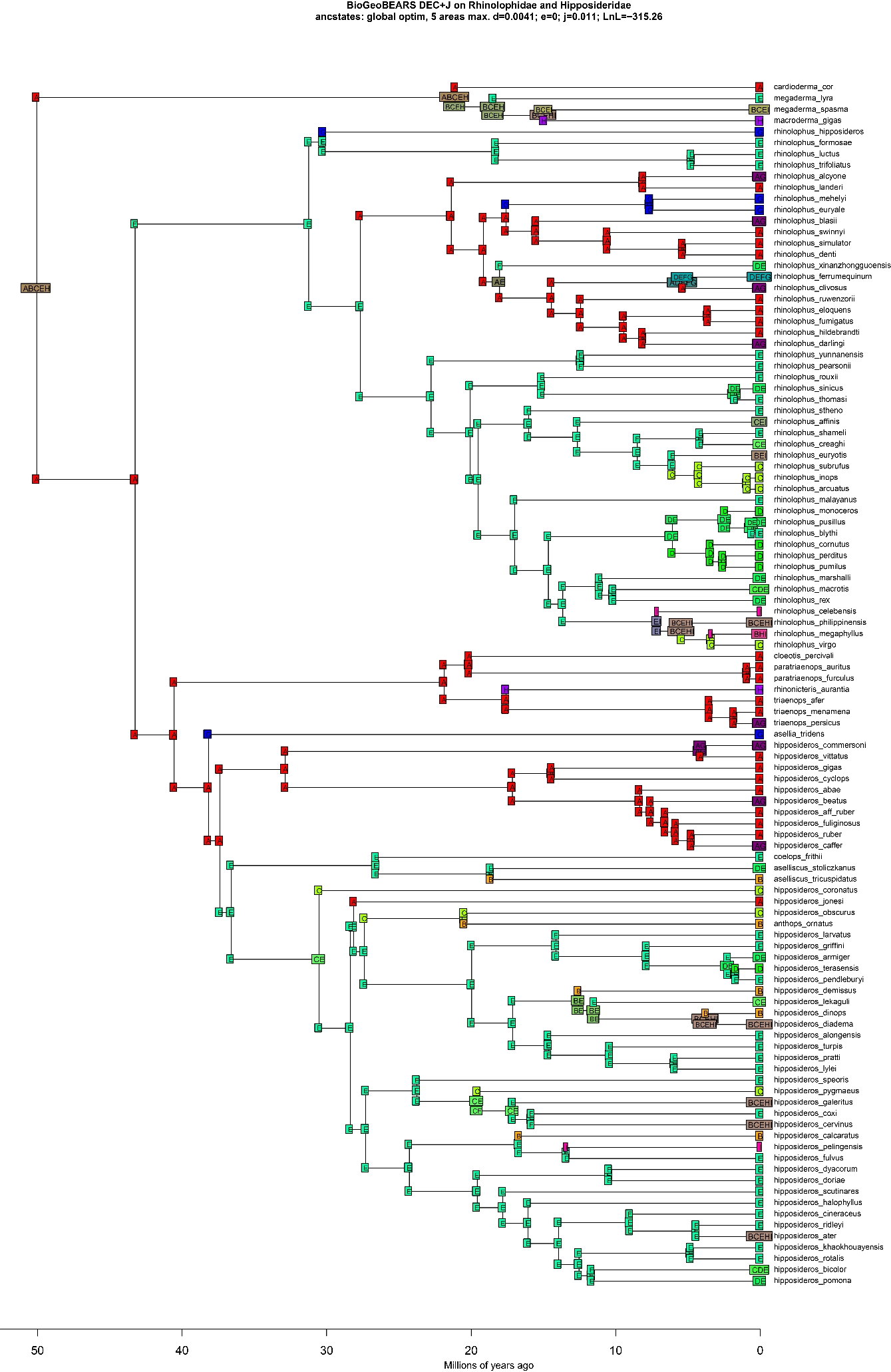


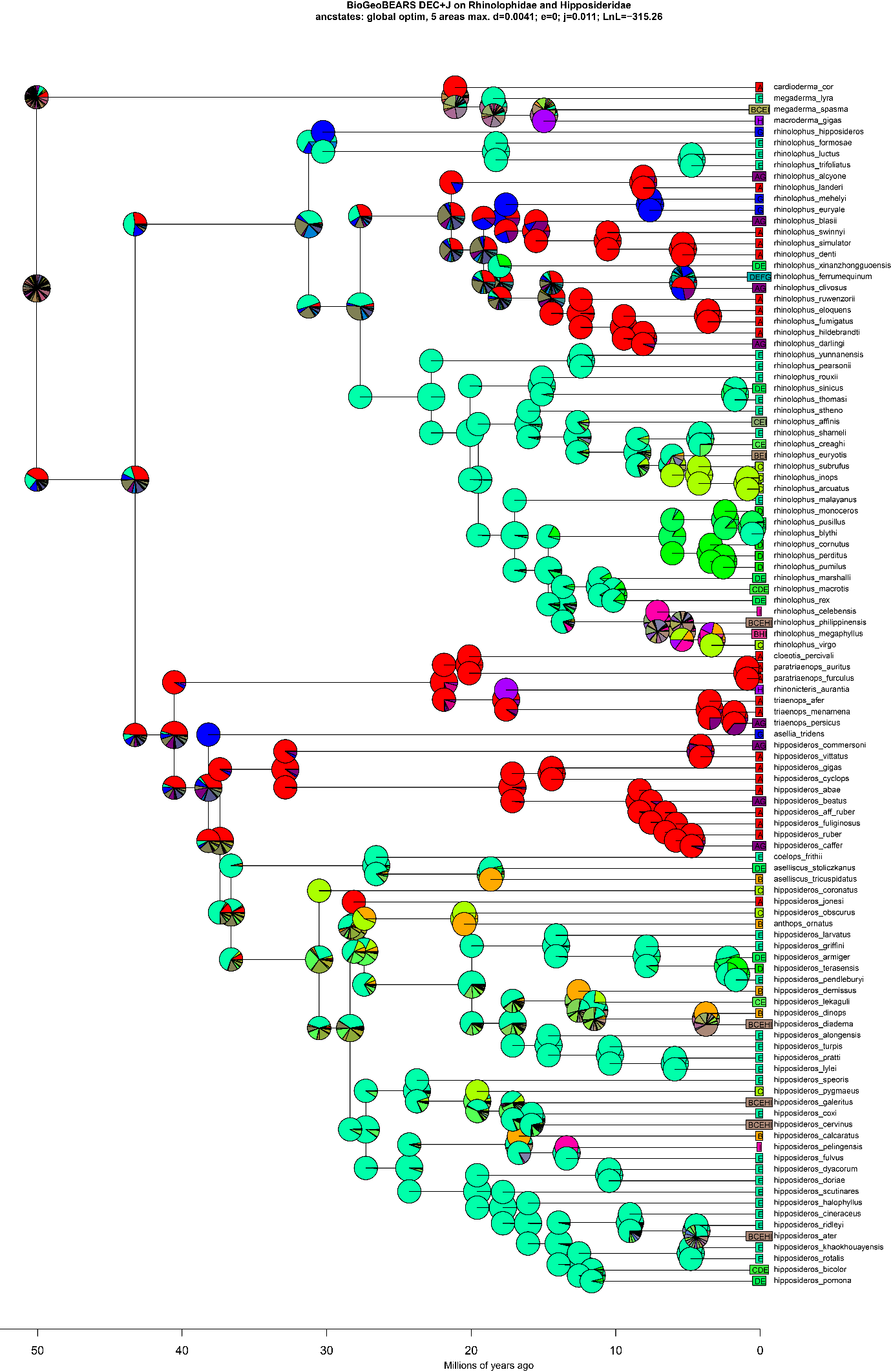


## **Supplementary Figure S4**


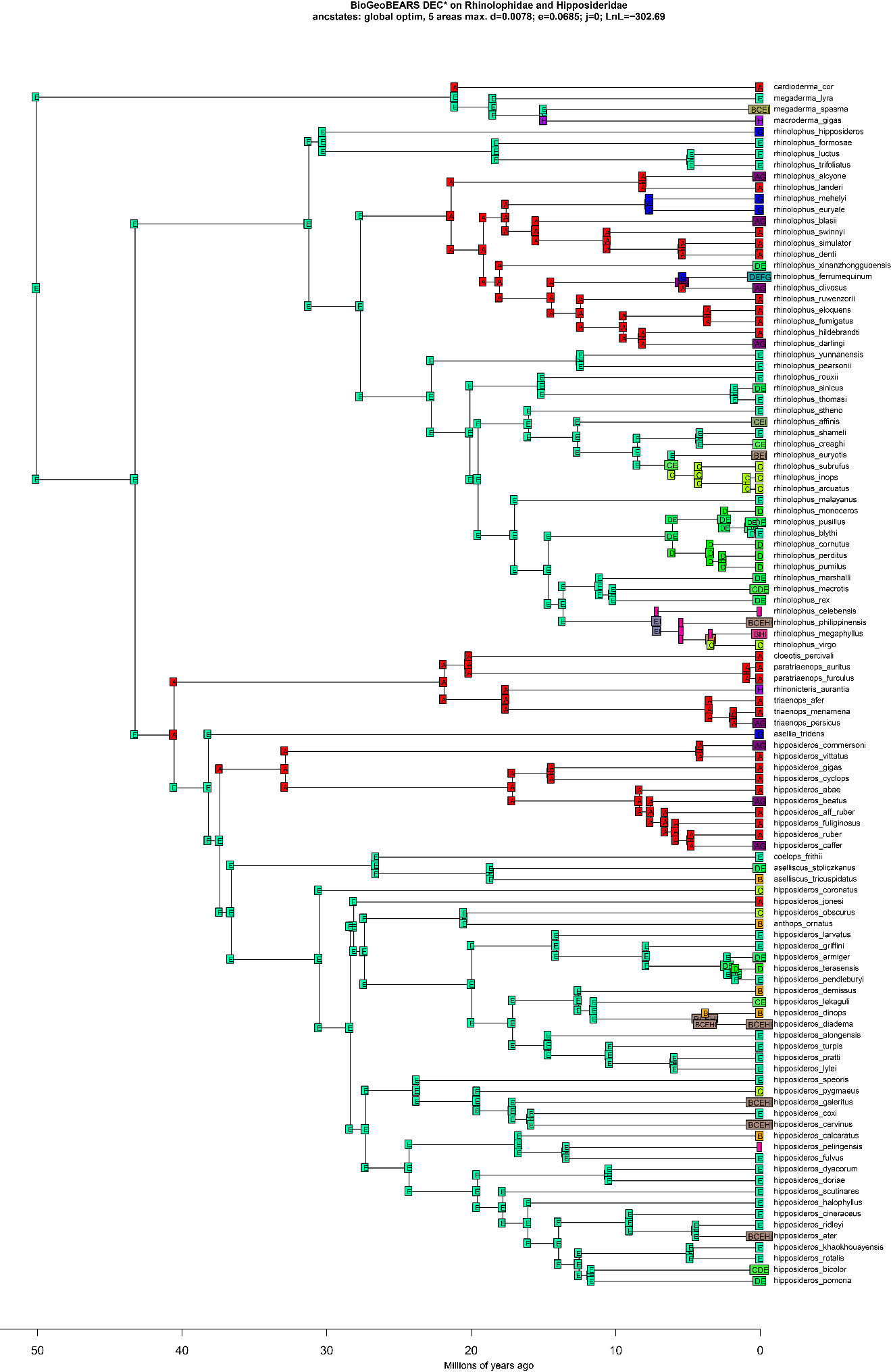


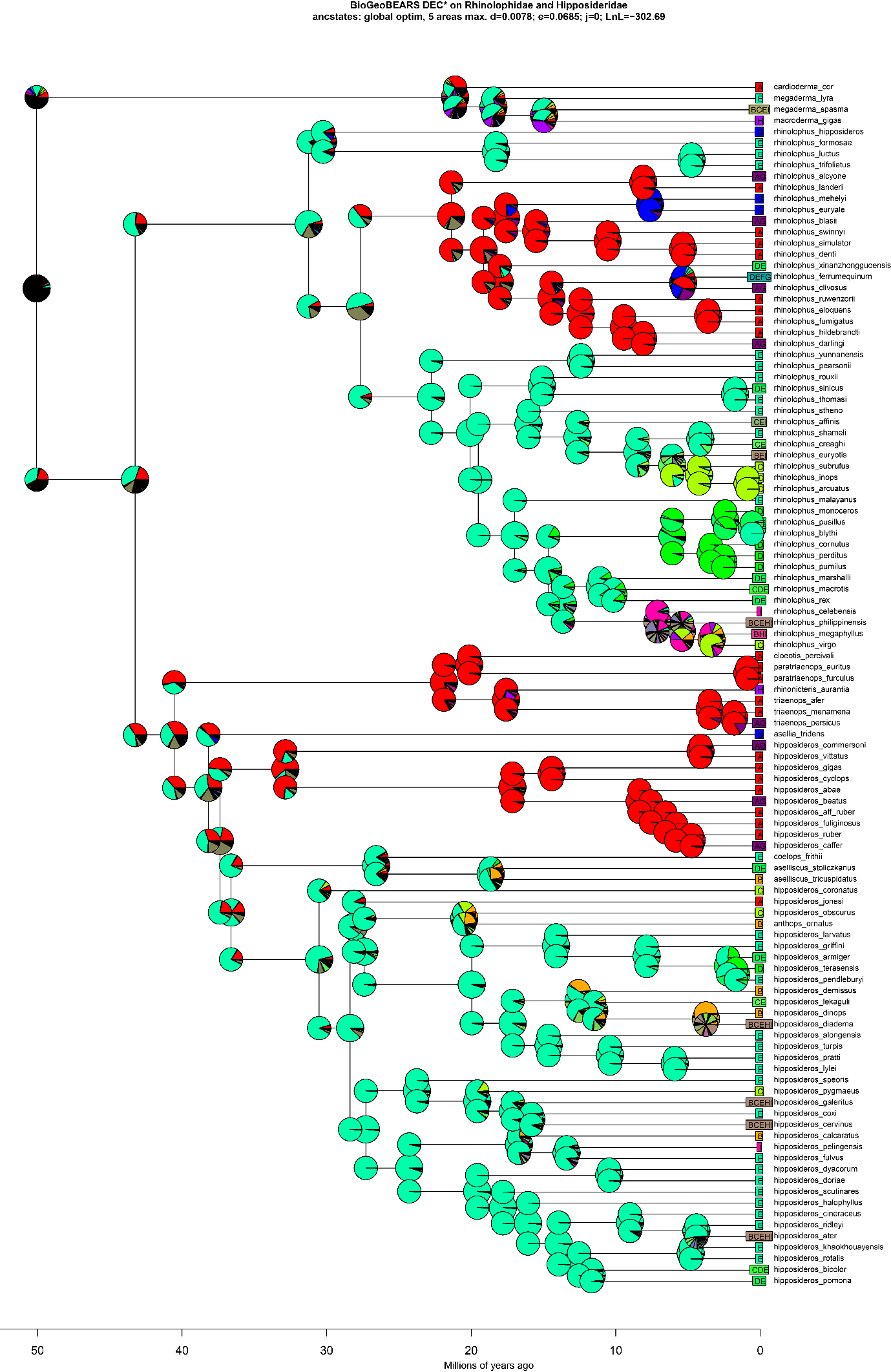


## **Supplementary Figure S5**


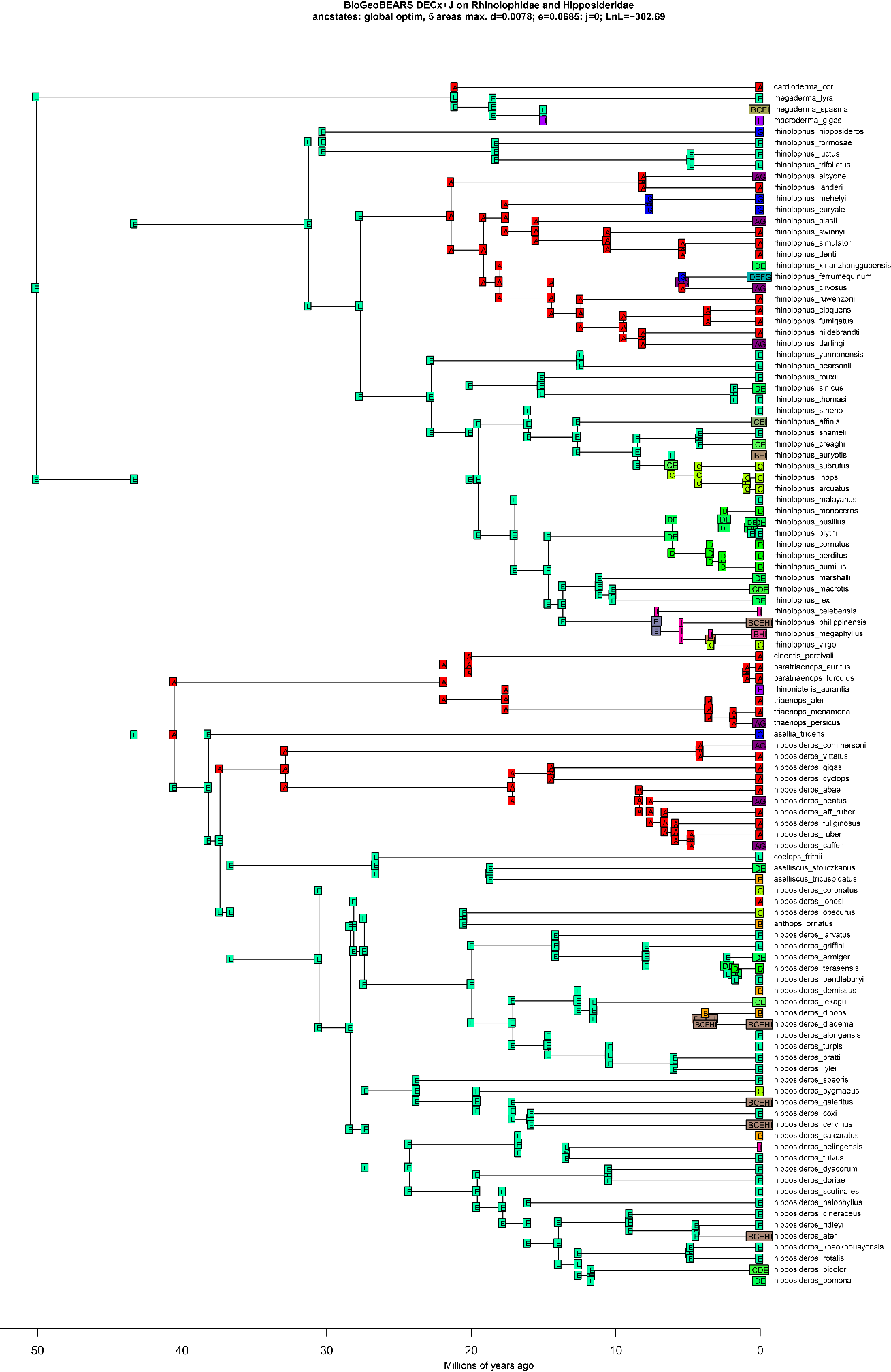


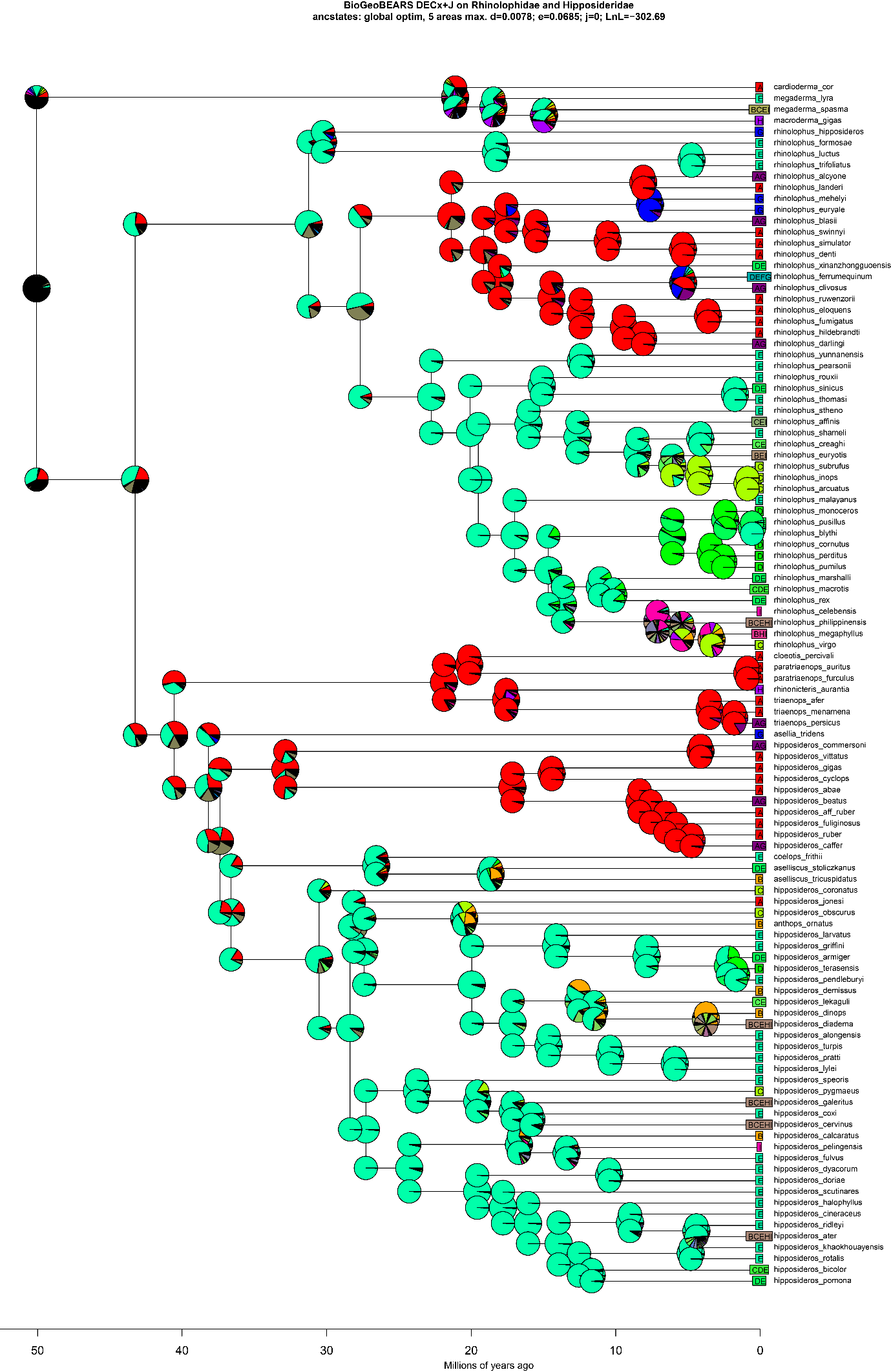


## **Supplementary Figure S6**


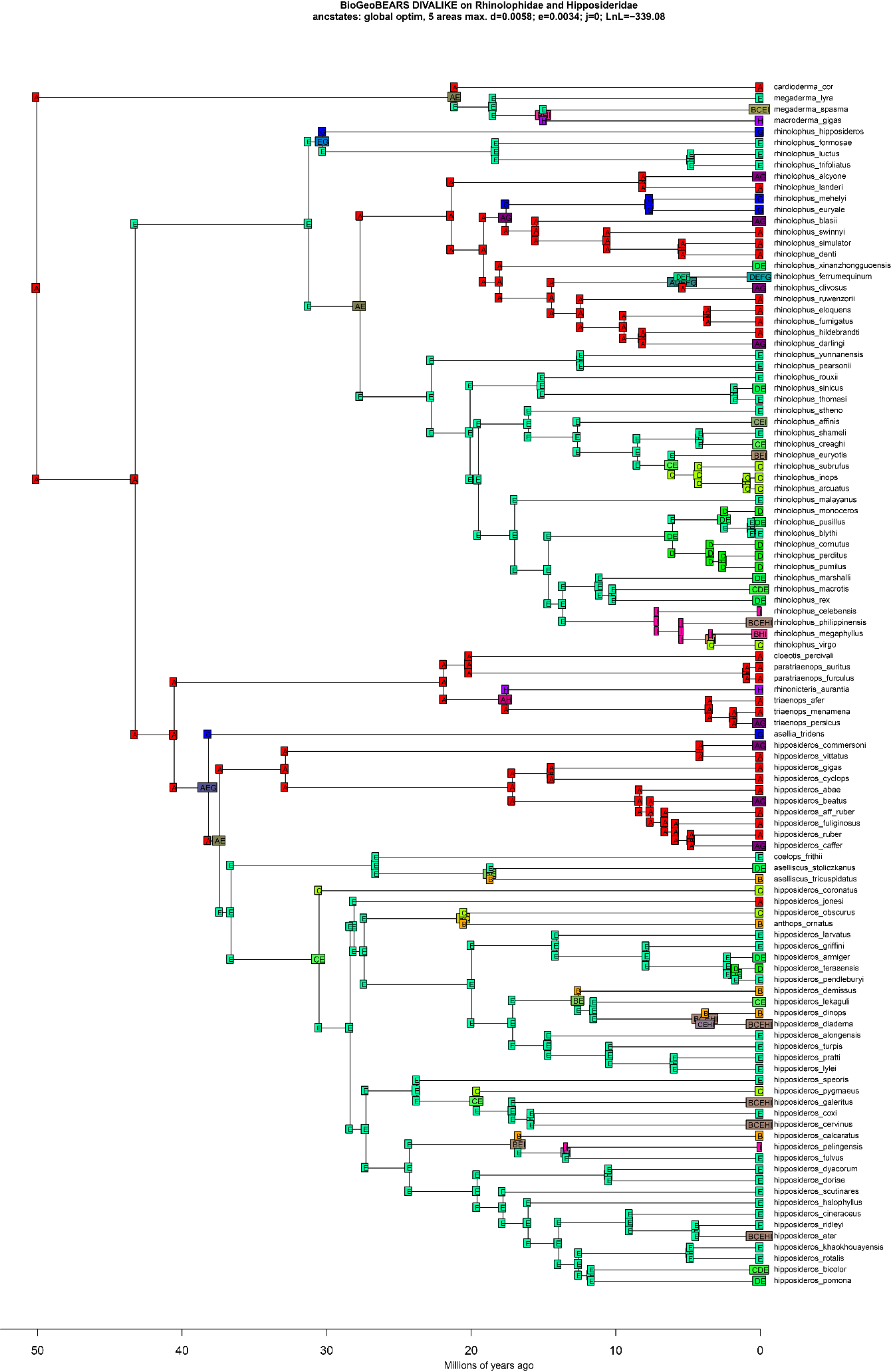


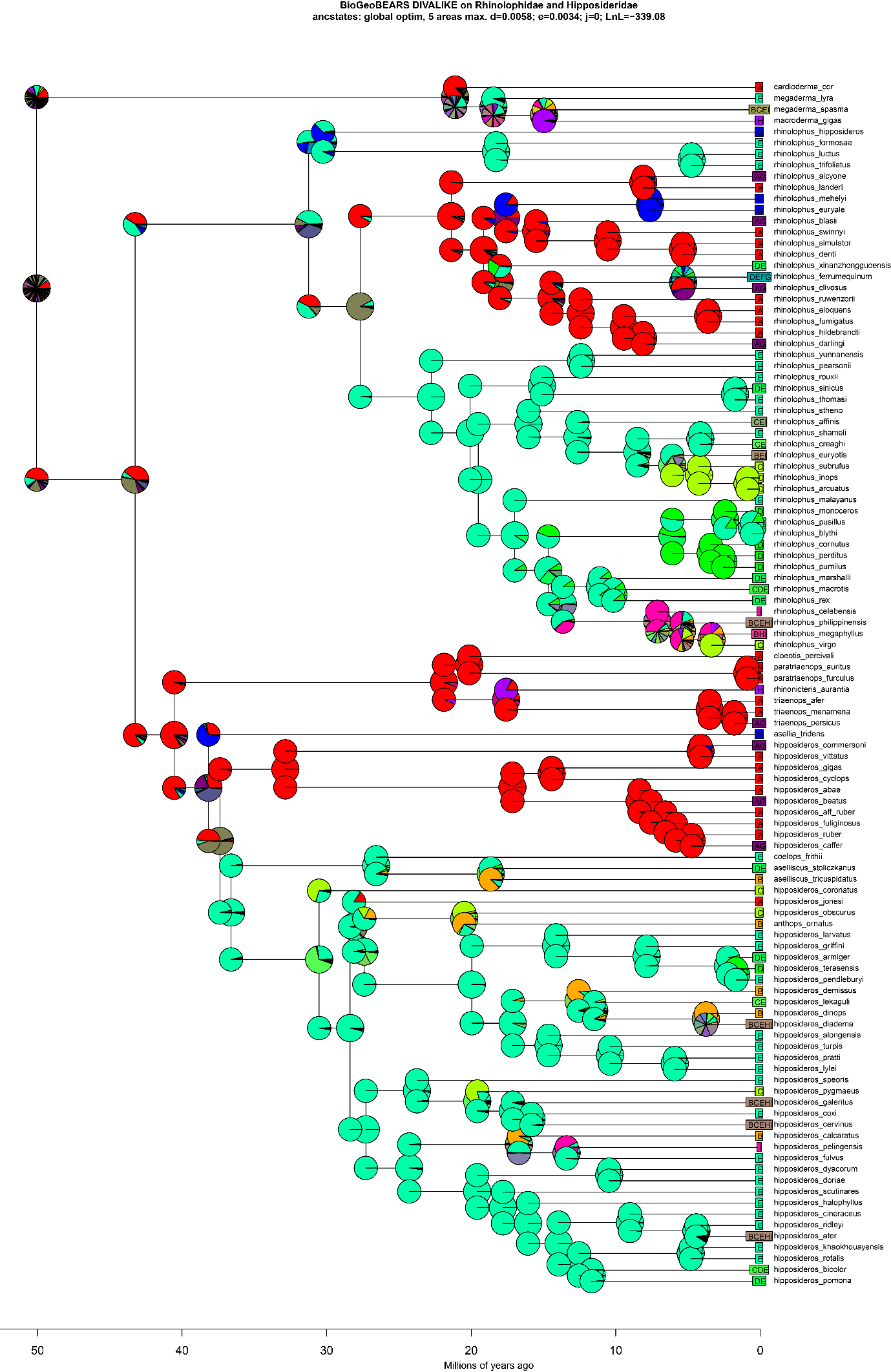


## **Supplementary Figure S7**


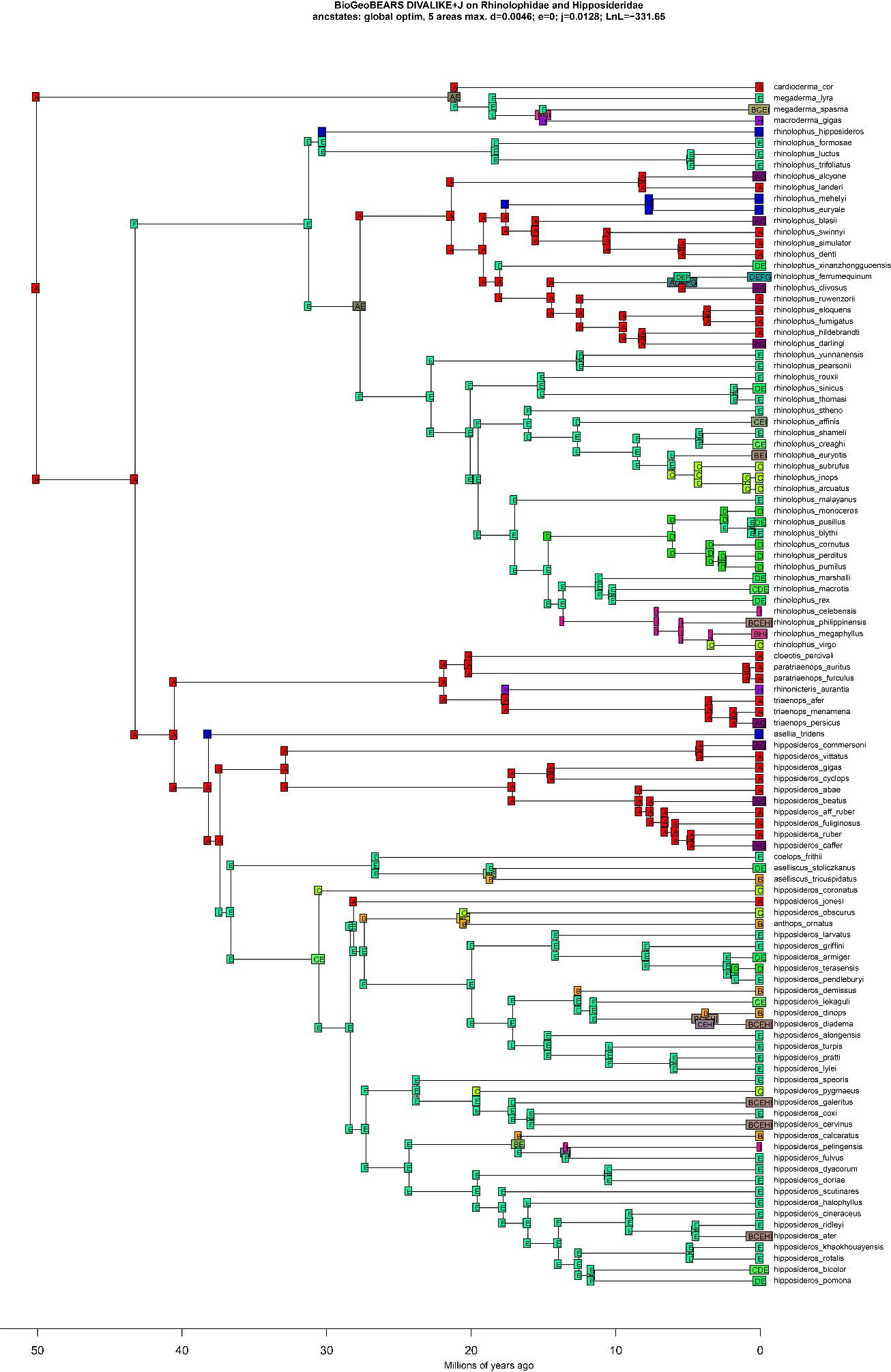


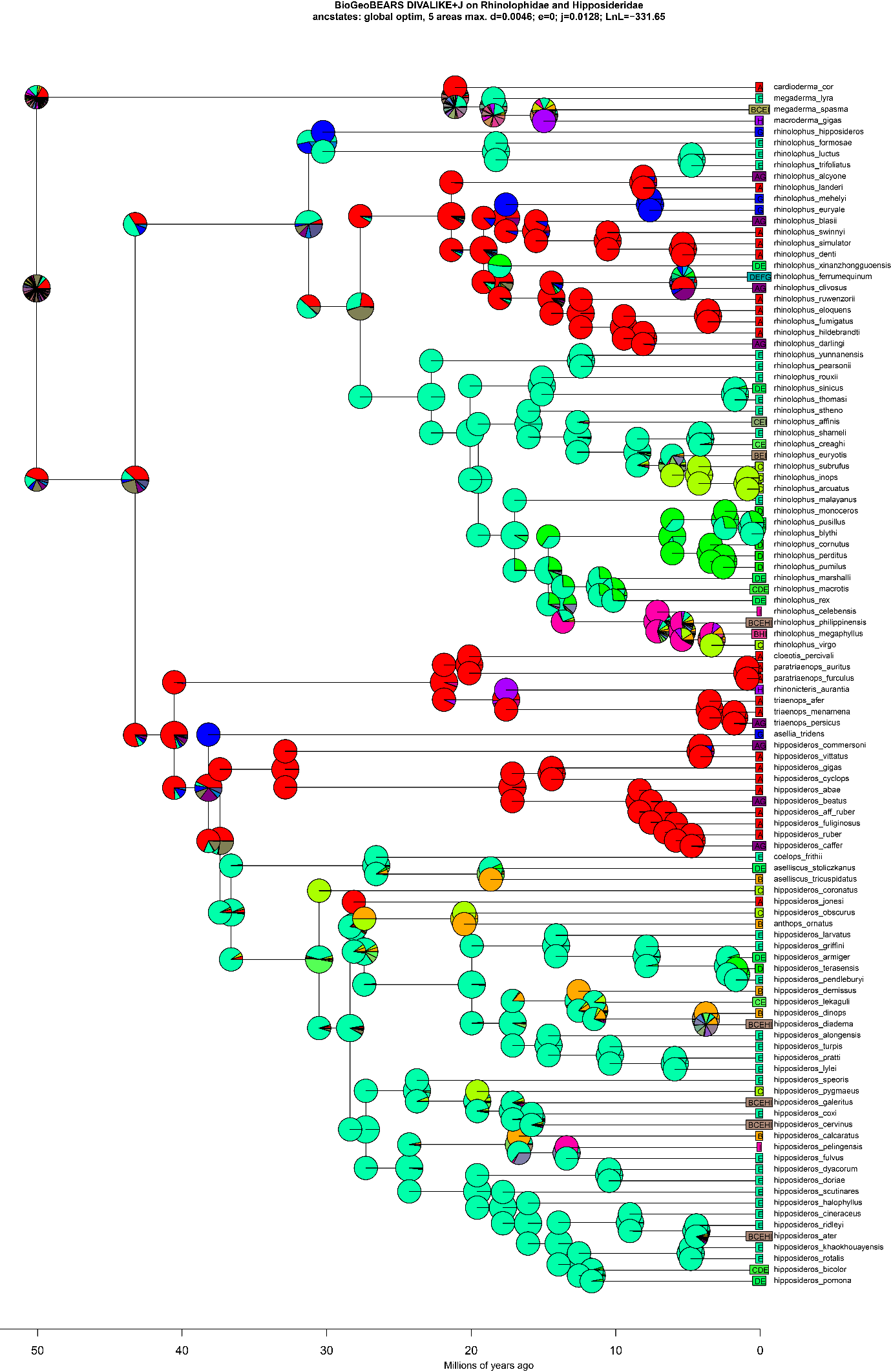


## **Supplementary Figure S8**


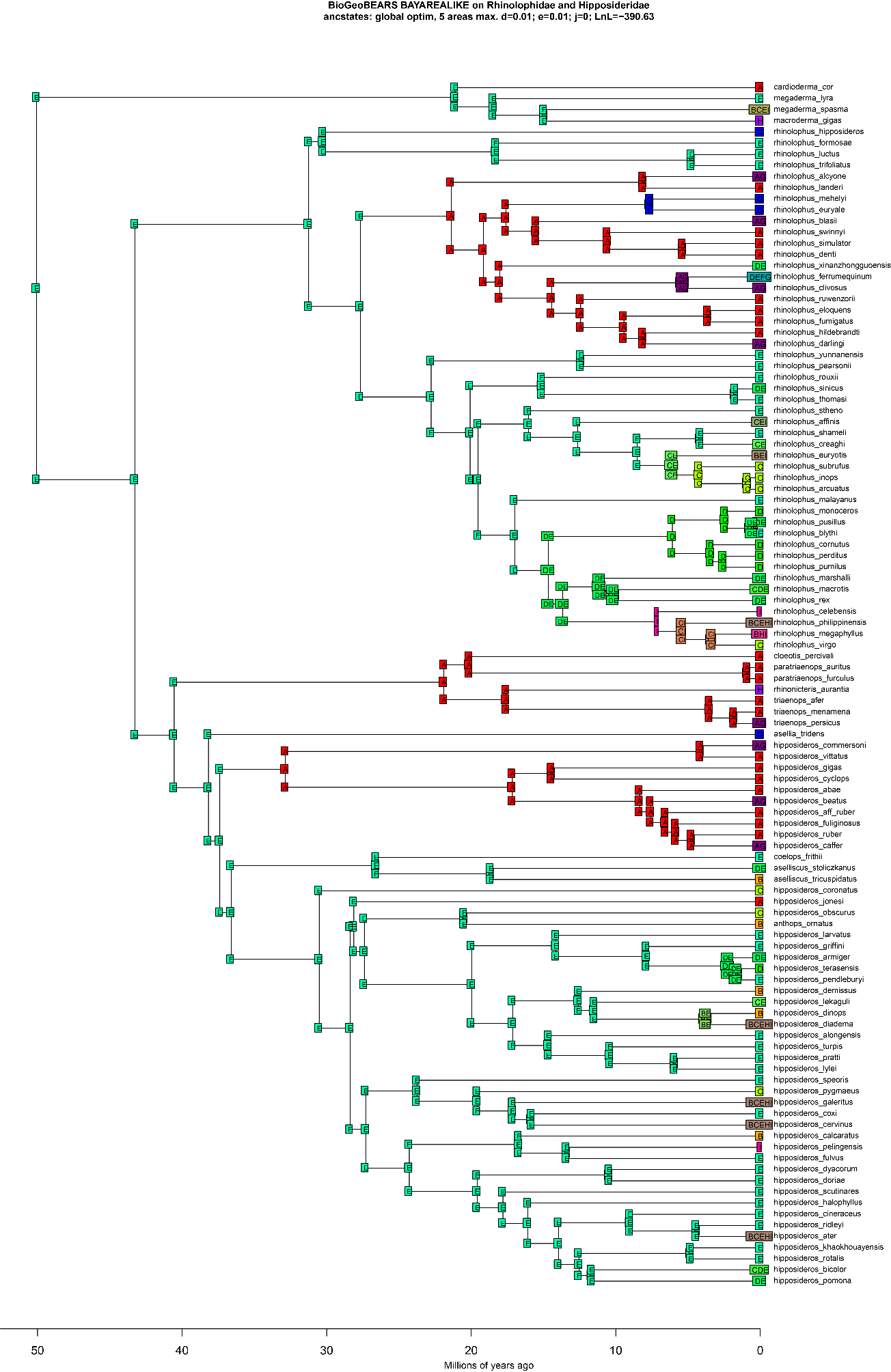


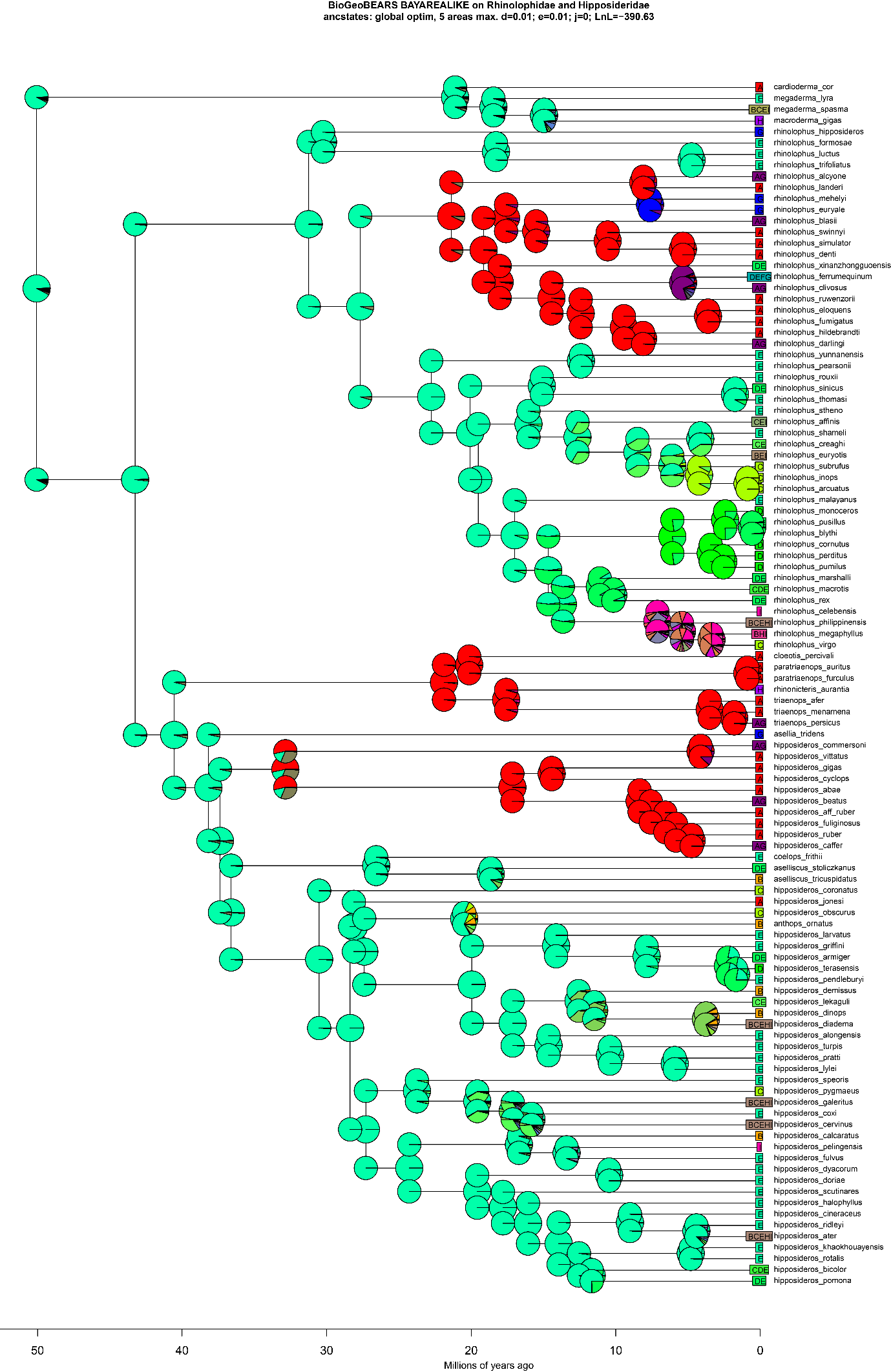


## **Supplementary Figure S9**


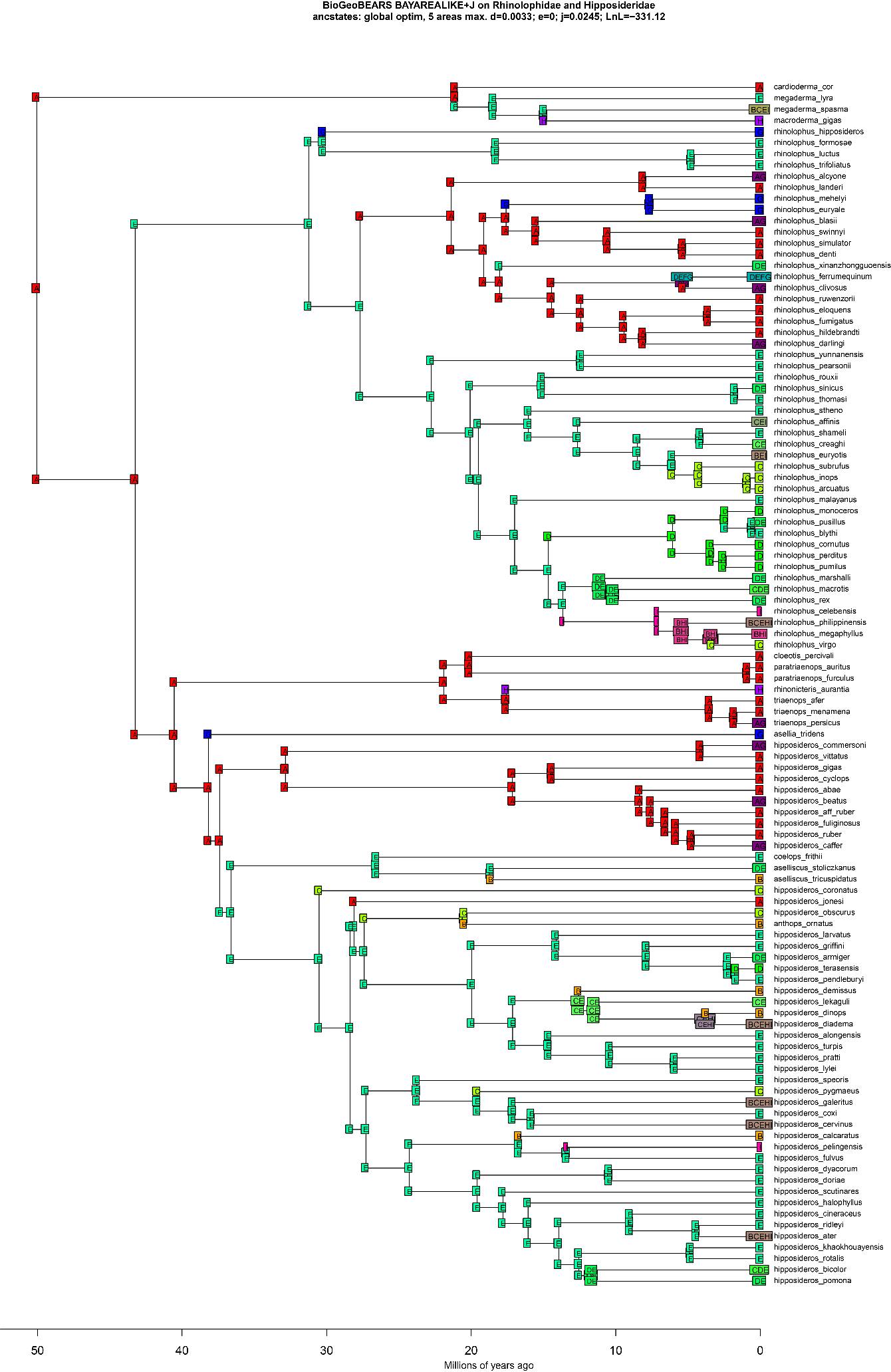


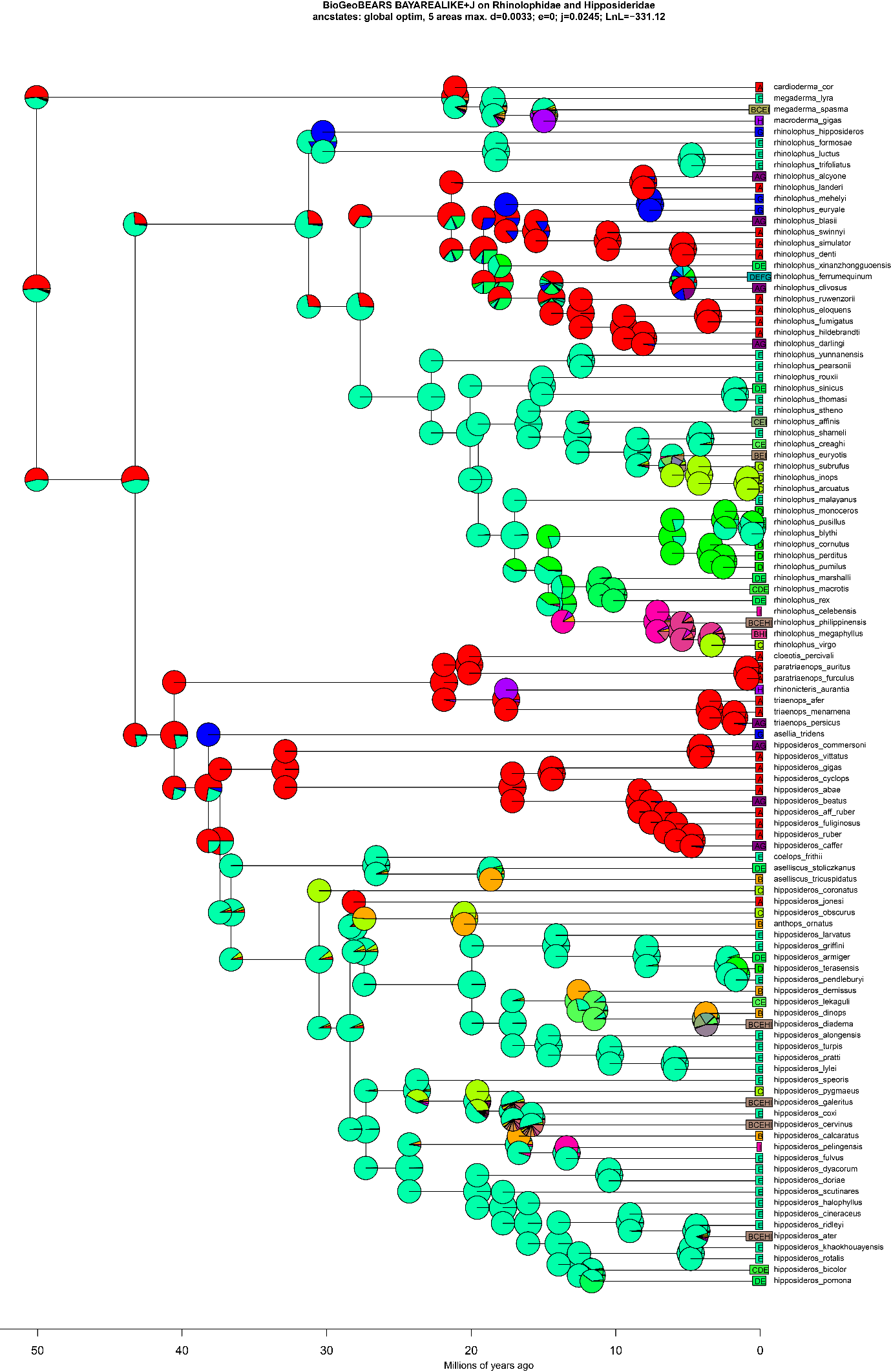

Supplement: Supplementary file 1 — Additional file 1. Supplementary figures. [file 12862_2022_2066_MOESM1_ESM.docx]
